# Label-Free Target Identification Reveals the Anticancer Mechanism of a Rhenium Isonitrile Complex

**DOI:** 10.3389/fchem.2022.850638

**Published:** 2022-03-14

**Authors:** Junhyeong Yim, Seung Bum Park

**Affiliations:** ^1^ Department of Biophysics and Chemical Biology, Seoul National University, Seoul, South Korea; ^2^ CRI Center for Chemical Proteomics, Department of Chemistry, Seoul National University, Seoul, South Korea

**Keywords:** anticancer agents, label-free target identification, metal-based drug, rhenium isonitrile complex, heat shock protein 60

## Abstract

Elucidation of the molecular mechanism of therapeutic agents and potential candidates is in high demand. Interestingly, rhenium-based complexes have shown a highly selective anticancer effect, only on cancer cells, unlike platinum-based drugs, such as cisplatin and carboplatin. These differences might be attributed to their different molecular targets. We confirmed that the target of tricarbonyl rhenium isonitrile polypyridyl (TRIP) complex is a protein, not DNA, using ICP-MS analysis and identified heat shock protein 60 (HSP60) as its target protein using a label-free target identification method. The subsequent biological evaluation revealed that TRIP directly inhibits the chaperone function of HSP60 and induces the accumulation of misfolded proteins in mitochondria, thereby leading to the activation of mitochondrial unfolded protein response (mtUPR)-mediated JNK2/AP-1/CHOP apoptotic pathway.

## Introduction

Cancer, the uncontrolled growth of cells, is the second leading cause of death in the United States (Shewach and Kuchta, 2009; Siegel et al., 2021). Various therapeutic agents have been developed to treat cancers (Thanarajasingam et al., 2018). Before the 1980s, chemotherapy focused on inhibiting the proliferation of cancer cells (Falzone et al., 2018); methotrexate inhibits the enzymes responsible for nucleotide synthesis, including dihydrofolate reductase and thymidylate synthase (Walling, 2006). Similarly, vincristine inhibits tubulin polymerization, leading to mitotic arrest (Bates and Eastman, 2017), and cisplatin crosslinks to DNA (Basu and Krishnamurthy, 2010), all resulting in the prevention of cell division. Unlike other drugs, cisplatin, a platinum-based compound, targets DNA, rather than proteins. Since platinum complex binds to DNA, various platinum analogs (e.g., carboplatin and oxaliplatin) were developed and used for cancer treatment to overcome the cisplatin-induced non-selective cytotoxic effects and resistance to treatments (Go and Adjei, 1999; Cox and Ang, 2009; Riddell and Lippard, 2018). Interestingly, the efficacy and side-effects of oxaliplatin are quite different from those of cisplatin and carboplatin (Machover et al., 1996; Rixe et al., 1996). Hemann and his co-workers revealed that oxaliplatin kills cancer cells by inducing ribosome biogenesis stress instead of DNA damage responses (Bruno et al., 2017). Subsequently, another study showed that oxaliplatin and some derivatives induce nucleolar stress derived from their organic ligands (Sutton et al., 2019), which means that the ligands of platinum-based complexes could alter their target proteins and modes of action. These events were also observed in non-platinum-based anticancer agents. For instance, ruthenium-based complexes target either DNA or proteins depending on their ligands (Ang et al., 2007; Gill et al., 2016). The types of organic ligands in titanium-based complexes can alter their targets. (Guo et al., 2000; Miller et al., 2020). Therefore, the ligand modification in metal-based complexes can significantly influence their engagement to different targets (Meggers, 2007; Blanck et al., 2012).

Recently developed rhenium-based compounds have shown anticancer effects (Knopf et al., 2017). Wilson and his co-workers produced various rhenium analogs via combinatorial synthesis and selected TRIP as a therapeutic candidate with improved anticancer efficacy (Konkankit et al., 2019a; Konkankit et al., 2019b; King et al., 2019). They ascertained that TRIP induced misfolded protein accumulation, mitochondrial fission, and C/EBP homologous protein (CHOP)-mediated apoptosis. Moreover, using a TRIP-resistant A2780 cell line, they revealed that TRIP resistance might be caused by the overexpression of metallothionein 1E (MT1E) gene, which is related to the detoxification of metal-based compounds (Marker et al., 2020). Even though there are high demands to reveal the exact molecular target of TRIP, the application of conventional target identification methods with affinity-based probes is quite limited since the structural modification of organic ligands in rhenium-based complexes might hamper the activity of its probes or alter the engagement pattern to their targets (Bregman et al., 2006; Feng et al., 2011). To circumvent these challenges, we hypothesized that label-free target identification could be a method of choice for identifying cellular targets of TRIP without structural modification. In this study, we identified and validated HSP60 as the target protein of TRIP *via* label-free target identification. We also revealed the molecular mechanism underlying the cytotoxicity of TRIP.

## Materials and Methods

### Cell Culture

HeLa (human cervical adenocarcinoma cell line), CaSki (human cervical epidermoid carcinoma cell line), A375P (human malignant melanoma), HEK293T (human embryonic kidney cell line), and C8-D1A (murine astrocyte cell line) were obtained from American Type Culture Collection (ATCC). HeLa (ATCC; CCL-2) and CaSki (ATCC; CRL-1550) were cultured in Roswell Park Memorial Institute (RPMI) 1,640 Medium (Gibco; #11875-093) supplemented with heat-inactivated 10% fetal bovine serum (FBS) (Gibco; #16000-044), and 1% penicillin (100 units/mL)/streptomycin (100 μg/mL) solution (Gibco; #15240-062). A375P (ATCC; CRL-3224), HEK293T (ATCC; CRL-3216), and C8-D1A (ATCC; CRL-2541) were cultured in Dulbecco’s Modified Eagle’s Medium (DMEM) (Gibco; #11995-065) supplemented with heat-inactivated 10% FBS (Gibco; #16000-044), and 1% penicillin/streptomycin solution (Gibco; #15240-062). All types of cells were maintained in 100-mm cell culture dishes in a humidified atmosphere of a 5% CO_2_ incubator at 37°C. All cells were cultured every 2 days using trypsin (Gibco; #12605-010).

### Cell Viability Assay

5,000 cells of HeLa, CaSki, A375P, and HEK293T were seeded per well at the transparent flat-bottom 96-well plate (Corning; #3598), and 10,000 cells of C8-D1A were seeded per well at the transparent flat-bottom 96-well plate and sealed with breathable sealing tape (Axygen; BF-400-S). After 24-h incubation, cisplatin (Sigma; P4393), carboplatin (Sigma; C2538), and TRIP were treated for 48 h. After changing the culture medium to 100 μL of fresh culture medium, 10 μL of Ez-cytox solution (Dogen; EZ-BULK150) was added to the 96-well plate. After 2-h incubation, absorbance at 455 nm was measured using a microplate reader (Biotek; Synergy).

### Inductively Coupled Plasma-Mass Spectrometry

5,000 cells/ml of HeLa were seeded in a 150-mm cell culture dish (Corning; #430599) and allowed to grow for 48 h. HeLa cells were then treated with 10 μM of either cisplatin (P4393; Sigma) or TRIP for 1, 2, 4, and 8 h. After washing with 1× phospho-buffered saline (PBS, Welgene; ML008-02), HeLa cells harvested by trypsinization (Gibco; #12605-010) were used for either protein or DNA extraction.

To measure metal accumulations in proteins, we re-suspended cells in 300 μL of distilled water (DW) and lysed by freeze and thaw cycle three times in liquid nitrogen. The cell lysates were clarified by centrifugation at 20,000 g, 4°C for 20 min. The concentration of the soluble protein fraction was measured by BCA (bicinchoninic acid) protein assay kit (Thermo; #23225). Cell lysates were digested in concentrated HNO_3_ at 160°C for 3 h and rested at room temperature overnight. The digested cell lysates were diluted in DW to make the final concentration of HNO_3_ less than 5%. Metal contents, platinum for cisplatin and rhenium for TRIP, were quantified with an ICP-MS (Perkin-Elmer SCIEX; NexION 350D) by measuring the most abundant isotope ^195^Pt and the most stable isotope ^185^Re, and calculated using a calibration curve from a series of concentrations of Pt and Re standards, respectively. The metal accumulations in proteins were expressed as ng Pt or Re/mg protein.

In order to quantify metal accumulations in DNA, cells were re-suspended in 300 μL of lysis buffer [100 mM NaCl, 25 mM EDTA (Acros; #11843-5000), 0.5% SDS (w/v) (Acros; #230420100), 0.1 mg/ml proteinase K (Merck; #70663-4CN), and 10 mM Tris-HCl, pH 8.0] and incubated at 50°C overnight. A 300 μL of phenol:chloroform:isoamyl alcohol mixture (25:24:1, Sigma; #77617) was added to cell lysates and shaken vigorously. After centrifugation at 13,000 g for 5 min, upper layer containing DNA, out of two layers, was transferred to a new tube. DNase-free dsRNase (Roche; # 11119915001) was added to DNA solution to a final concentration 1 μg/ml and incubated at 37°C for 1 h. Then, 200 μL of 7.5 M ammonium acetate (Merck; # 1.01116.1000) was added to DNA solution, followed by 400 μL of 100% ethanol (Daejung; #4023-4104), and the mixture was incubated at −80°C for 30 min. To precipitate DNA, the solution was centrifuged at 10,000 g for 5 min, and the precipitated DNA pellet was rinsed with 70% ethanol and dried by air. The DNA was dissolved in 50 μL of DW and added 100 μL of 8 mM NaOH solution (Acros; AC124260050). DNA concentration was quantified by measuring the absorbance at 260 nm. DNA was digested in concentrated HNO_3_ at 160°C for 3 h and rested at room temperature overnight. Metal contents in DNA were quantified by ICP-MS as same method as measuring metal contents in protein. The metal accumulations in DNA were expressed as ng Pt or Re/mg DNA.

### TS-FITGE

Procedures for TS-FITGE were previously described (Park et al., 2017). Briefly, HeLa cells were treated with either vehicle (veh) or 10 μM of TRIP for 4 h. Veh- or TRIP-treated HeLa cells were heat-denatured for 3 min at various temperature ranges, followed by 25°C for 3 min. The heated cells were washed with 1× PBS (Welgene; ML008-02) and re-suspended in the lysis buffer [PBS containing 0.4% IGEPAL CA-630 (Sigma; I8896) and 1× Protease Inhibitor Cocktail (PIC, Roche; #11873580001)]. Cells were lysed by freeze and thaw cycle three times in liquid nitrogen. The cell lysates were clarified by centrifugation at 20,000 g, 4°C for 20 min. The concentration of the soluble protein fraction was measured by BCA protein assay kit (Thermo; #23225). Then, each 50 μg of the protein was precipitated by cold acetone at −20°C for 20 min, followed by centrifugation at 20,000 g, 4°C for 7 min. After acetone washing three times, the protein pellet was re-suspended with 10 μL of conjugation buffer [7 M urea (Acros; #32738), 2 M thiourea (Acros; #13891), 4% (w/v) CHAPS (Sigma; C3023), and 30 mM Tris-HCl, pH 8.0]. Veh- and TRIP-treated proteins were added 1 μL of 0.4 mM Cy3-*N-*hydroxysuccinimide (NHS) and Cy5-NHS, respectively, and incubated at 4°C for 45 min. The dye-conjugated proteomes were precipitated by cold acetone at −20°C for 20 min, followed by centrifugation at 20,000 g, 4°C for 7 min. After acetone washing three times, the pellets were re-suspended with 50 μL of re-hydration buffer [7 M urea, 2 M thiourea, 2% (w/v) CHAPS, 40 mM DTT (Millipore; #1.11474.0025), and 1% pH 3–10 IPG buffer (Cytiva; #17-6000-87)]. The same amount of veh- and TRIP-treated samples were mixed and loaded on a pH 3–10, 24-cm Immobiline Drystrip gel (Cytiva; #17-6002-44). The primary dimension was separated by an isoelectric focusing system (Cytiva; Ettan IPGphor 3), and the secondary dimension was separated by a polyacrylamide gel electrophoresis (PAGE) system (Cytiva; Ettan DALTsix). The 2-dimensional (2D) gel was scanned with a fluorescent gel scanner (Azure Biosystems; Sapphire).

### In-Gel Digestion and Mass Spectromety

As previously described, the protein spots from silver-stained gels were excised, de-stained, and digested only with trypsin (Choi et al., 2018). The resulting peptides were identified by peptide sequencing using nanoAcquity UPLC-ESI-Q-TOF mass spectrometry (Waters; SYNAPT G2-Si HDMS). Peptides were eluted with a linear gradient of 5–40% buffer B [ACN/formic acid; 100:0.1 (v/v)] with buffer A [water/formic acid; 100:0.1 (v/v)] over 80 min, and MS scan cycle was composed of one MS scan followed by MS/MS scans of the 10 most abundant ions in each MS scan. The resulting MS data were converted to peaklist files (.pkl) using Protein Lynx Global Server (PLGS) 2.3 data processing software (Waters). Peaklists were searched using a global search engine Mascot (version 2.2.0) with the protein sequence database SwissProt (version 51.6, 257964 entries). The taxonomy filter for 2D-PAGE samples was *Homo sapiens* (human). The maximum number of one missed cleavage was permitted, and no fixed modifications were considered. As variable modifications, carbamidomethylation of Cys, oxidation of Met, phosphorylation of Ser or Thr, acetylation and formylation of Lys, *N-*terminal pyroglutamylation of Gln and Glu, and acrylamide adduct propionamide of Cys were considered for the identification of protein spots.

### Immunoblotting

HeLa cells were harvested and lysed in modified radioimmunoprecipitation assay (RIPA) buffer [150 mM NaCl, 1% IGEPAL CA-630 (Sigma; I8896), 0.5% deoxycholate (Sigma; #30970), 5 mM NaF (Sigma; S7920), 2 mM Na_3_VO_4_ (Sigma; # 450243), 1× PIC (Roche; #11873580001), and 50 mM Tris–HCl, pH 7.8] for 20 min on ice, and the cell lysates were clarified by centrifugation at 20,000 g, 4°C for 20 min. The concentration of the soluble protein fraction was measured by BCA protein assay kit (Thermo; #23225). Proteome was added with 5× SDS (Biosesang; SF 2002-110-00) and heated at 95°C for 5 min.

Equal amounts of the proteome were analyzed by SDS-PAGE and transferred to PVDF membranes (Bio-Rad; BR162-0177). The membranes were blocked with 2% bovine serum albumin (BSA, MP Biomedicals; #0216006980) in Tris-buffered saline containing 0.1% Tween-20 (TBS-T, Sigma; P9416) at room temperature for 1 h. To detect the desired proteins, the membranes were incubated overnight at 4°C with primary antibodies—1:1,000 dilution of HSP60 (Santa Cruz; sc-1052), eIF2α (Santa Cruz; sc-133132), p-eIF2α (phospho S51, Abcam; ab32157), ATF4 (CST; #11815), JNK (CST; #9252S), p-JNK (CST, #9251S), CHOP (CST, #2895); 1:2,000 dilution of GAPDH (CST; #2118), and β-actin (CST, #4970). After washing with TBS-T, the resulting membranes were exposed to HRP-conjugated secondary antibody at room temperature for 1 h—1:5,000 dilution of anti-rabbit (CST; #7074), anti-mouse (CST; #7076), and anti-goat (Santa Cruz; sc-2033). After washing with TBS-T, the membranes were incubated with enhanced chemiluminescence (ECL) prime kit (Cytiva; RPN2232). Chemiluminescent signals from desired proteins were detected using ChemiDoc (Bio-Rad).

### Cellular Thermal Shift Assay

HeLa cells were treated with either vehicle or 10 μM of TRIP for 4 h. Veh- or TRIP-treated HeLa cells were heat-denatured for 3 min at various temperature ranges, followed by 25°C for 3 min. The heated cells were washed with 1× PBS (Welgene; ML008-02) and re-suspended in the lysis buffer [PBS containing 0.4% IGEPAL CA-630 (Sigma; I8896) and 1× PIC (Roche; #11873580001)]. Cells were lysed by freeze and thaw cycle three times in liquid nitrogen. The cell lysates were clarified by centrifugation at 20,000 g, 4°C for 20 min. The soluble protein fraction was added with 5× SDS (Biosesang; SF 2002-110-00), heated at 95°C for 5 min, and subjected to immunoblotting.

### Preparation of Recombinant HSP60

HSP60 with *C-*term His-tag plasmid (Sino Biological; HG11322-CH) was transferred to BL21 (DE3) competent *E. coli* (Enzynomics; CP110). The BL21 cells were grown in 250 ml of LB broth (Biosesang; LR3004-250-02) at 37°C with shaking until OD_600_ reached 0.6. The cultures were then induced with the final concentration 0.4 mM IPTG (Sigma; I5502) at 37°C for 4 h. Cells were harvested by centrifugation at 5,000 g, 4°C for 10 min, and re-suspended by the protein buffer (150 mM NaCl, 1 mM DTT (Millipore; #1.11474.0025), and 10 mM Tris-HCl, pH 8.0) containing 1× PIC (Roche; #11873580001). Cells were lysed by sono-smasher (Ulsso Hitech; ULH-700S) and clarified by centrifugation at 13,000 g, 4°C for 30 min. Cell lysates were incubated with Ni-NTA agarose (Qiagen; #30210) at 4°C for 1 h and purified by various concentrations of imidazole (Sigma; I5513) in the protein buffer. Imidazole was removed by buffer exchange with 10K Amicon (Millipore, UFC501096), and the concentration of purified HSP60 was measured by BCA protein assay kit (Thermo; #23225). The purity of HSP60 was determined by SDS-PAGE with the final purity over 90% in Coomassie staining (Abcam; ab119211).

### Surface Plasmon Resonance Assay

SPR experiments were performed using a real-time biomolecular interaction analysis system (Cytiva; Biacore T100). Recombinant HSP60 proteins were immobilized to a CM5 sensor chip (Cytiva; BR100012) with amine coupling kit (Cytiva; BR100050) in 1× PBS (Welgene; ML008-02) containing 0.005% Tween 20 (Sigma; P9416) at pH 4.0 acetate buffer (Cytiva; BR100349). Final immobilization level of HSP60 reached 1,881 RU. To monitor the biophysical interaction between HSP60 and TRIP, we injected various concentrations of TRIP in the running buffer [PBS (pH 7.4) containing 0.005% Tween 20] for 60 s with a flow rate of 30 μL/min. The dissociation event of TRIP from HSP60 was monitored by injecting the running buffer for 300 s at 25°C. Data were analyzed to calculate kinetic parameters by fitting sensorgrams with the one-on-one binding model using Biacore T100 Evaluation software (Cytiva).

### Differential Scanning Fluorimetry Assay

DSF experiments were carried out in Step One Real-Time PCR system (Applied Biosystems; #4376357). 4 μM of the recombinant HSP60 proteins was incubated with 5,000× Syprox-Orange (Sigma; S5692) in the absence or presence of TRIP for 1 h, followed by heat scanning from 25°C to 95°C in an increment of 1% continuous temperature. Normalized and first derivative reporter data were analyzed.

### Heat Shock Protein 60/Heat Shock Protein 10 Refolding Assay

The refolding activity of HSP60 was measured by a human HSP60/HSP10 protein refolding kit (R&D Systems; K-300) according to the manufacturer’s protocol. Briefly, luciferase protein was denatured by heat shock at 45°C for 7 min on C1000 Touch Thermal Cycler (Bio-Rad; #1841000) and incubated with the HSP60/HSP10 complex in the absence or presence of various concentrations of either TRIP or epolactaene *t-*butyl ester (ETB), a known HSP60 inhibitor, at 30°C for 1.5, 3, and 6 h on C1000 Touch Thermal Cycler. After that, the refolded luciferase protein was mixed with luciferin to measure the chaperone activity of HSP60 on the basis of luminescence signals. The luminescence signals were measured using a microplate reader (Biotek; Synergy HTX).

### siRNA Transfection

For HSP60 knockdown experiments, a short interfering RNA (siRNA) duplex against HSP60 was used (Bioneer; #3329). siRNA oligonucleotides were transfected in HeLa cells for 2 days using Lipofectamine RNAiMAX (Thermo; #15338100) and Opti-MEM (Gibco, #31985070) based on manufacturer’s instructions. For the experiments to assess the activity change of TRIP after gene knockdown, cells were transfected with siRNA for 2 days and treated with either cisplatin or TRIP for another 2 days, followed by a cell viability assay.

SiRNA Oligonucleotide sequences for HSP60 are as follows:

SiHSP60-1 sense 5′- GUG UUG AAG GAU CUU UGA UTT -3′

antisense 5′- AUC AAA GAU CCU UCA ACA CTT -3′

SiHSP60-2 sense 5′- GAA GUU UGA UCG AGG CUA UTT -3′

antisense 5′- AUA GCC UCG AUC AAA CUU CTT -3′

SiHSP60-3 sense 5′- CAG UGU ACU GCU UUC AAU UTT -3′

antisense 5′- AGU UGA AAG CAG UAC ACU GTT -3′

### Flow Cytometry Analysis

HeLa cells were treated with each compound for the designated time and dose, or transfected with siRNAs using Lipofectamine RNAiMAX (Thermo; #15338100). The cells were trypsinized and re-suspended in PBS containing 2% FBS (Gibco; #16000-044). To measure mitochondrial reactive oxygen species (ROS) and polarization, we stained the same number of re-suspended cells with either 2.5 μM of mitoSOX (Invitrogen; M36008) or 500 nM of TMRE (tetramethylrhodamine ethyl ester) (Invitrogen; T669) at 37°C for 1 h. To measure apoptosis, we also stained the same number of re-suspended cells with Annexin V-FITC Apoptosis Staining and Detection Kit (Abcam; ab14085) according to the manufacturer’s protocol. The stained cells were subjected to flow cytometry analysis using FACSAria II (BD).

### Reporter Gene Assay

HeLa cells were seeded 10,000 cells per well at a white 96-well plate (Falcon; #353296). HeLa cells were transfected with a 1 to 1 ratio of 3× AP1pGL3 (Addgene; #40342) and pRL-TK (Promega; E2241) mixture using LTX plus (Invitrogen; #15338100) and Opti-MEM (Gibco, #31985070) according to the manufacturer’s protocol and incubated for 24 h. The transfected cells were treated with 10 μM of cisplatin (Sigma; P4393), 10, 20, 40 μM of TRIP, or 200 nM of thapsigargin (Sigma; T9033) for 24 h. To measure the AP-1 activation, cells were washed with 1× PBS (Welgene; ML008-02) and lysed with 20 μL of 1× passive lysis buffer for 15 min. Luciferase signals were measured by dual-luciferase reporter assay system (Promega; E1980) using a microplate reader (Biotek; Synergy HTX). Expression levels of AP-1 were normalized by the Renilla luciferase signal.

### Mitochondrial Monitoring in Live Cell

25,000 cells of HeLa were seeded per well on an 8-well chamber (Nunc; #155409). To monitor mitochondrial fission, we stained the mitochondria of HeLa cells with 100 nM of Mito-tracker deep red (Invitrogen; M22426) and the nuclei of HeLa cells with 4 nM of Hoechst 33342 (Thermo; #62249) for 30 min. The stained cells were pre-treated with 50 μM of Mdivi-1 (Sigma; M0199) for 1 h, followed by 10 μM of TRIP treatment. HeLa cells were monitored in a humidified atmosphere of 5% CO_2_ at 37°C using a high-resolution microscope (Cytiva; Delta Vision).

### Statistical Analysis

Statistical analyses of all experiments were performed with Student’s t-test using Graph Pad Prism (GraphPad). Data are represented as mean ± SD (standard deviation), as indicated in the individual figure legends. The *p*-value is stated in the legend of each figure.

## Result and Discussion

### Tricarbonyl Rhenium Isonitrile Polypyridyl Induces Selective Death of Cancer Cells *via* Working on Protein, not DNA

**FIGURE 1:**
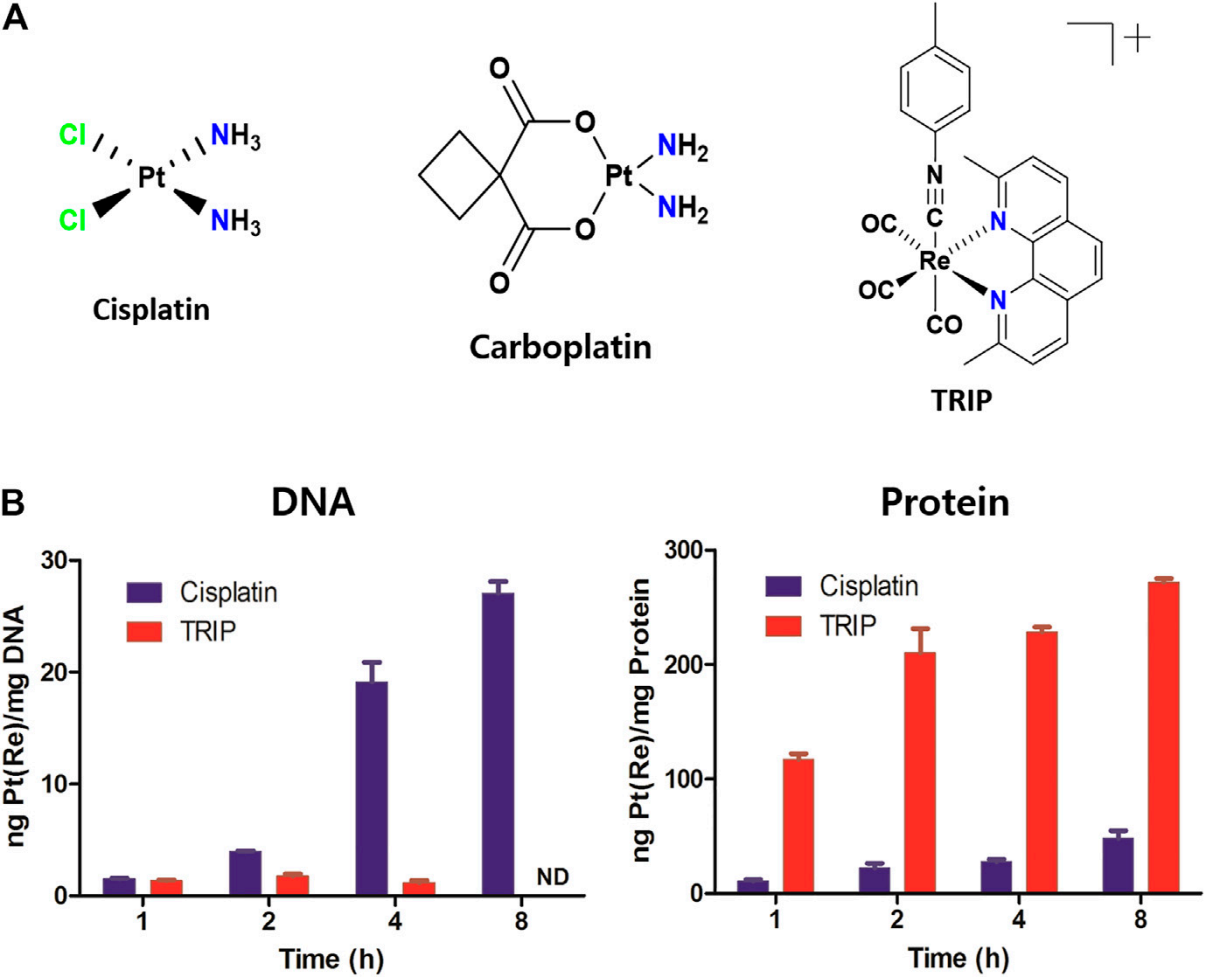
Each metal-based anticancer agent showed distinct accumulation in DNA and proteins. **(A)** Chemical structures of metal-based anticancer agents. **(B)** HeLa human cervical cancer cells were treated with either cisplatin or TRIP for 1, 2, 4, and 8 h. Platinum and rhenium were quantitatively measured by ICP-MS for cisplatin and TRIP, respectively.

Although anticancer therapeutic agents focus on inhibiting the aberrant proliferation of cancer cells, they unintentionally affect the fast-growing normal cells as well, resulting in anemia (Groopman and Itri, 1999), alopecia (Chon et al., 2012), and fertility problems (Blumenfeld, 2012). Interestingly, the growth inhibition assay ascertained that although cisplatin and carboplatin kill all cell lines, TRIP selectively kills cancer cells, not normal cells (Figure 1A, Supplementary Figure S1, Table 1), indicating that the mechanism of TRIP might be different from that of platinum-based drugs. It was hypothesized that the non-selective cytotoxicity of platinum-based compounds might be correlated with the efficiency of their cellular uptake and platinum-DNA adduct formation (Dasari and Tchounwou, 2014). To verify whether TRIP generates metal-DNA adducts like cisplatin, we conducted ICP-MS (inductively coupled plasma-mass spectrometry) analysis. HeLa human cervical cancer cells were treated with 10 μM of either cisplatin or TRIP for 1, 2, 4, and 8 h. The accumulation of metal (platinum for cisplatin and rhenium for TRIP) was quantitatively compared between proteins and DNA. Cisplatin treatment induced the DNA platination in a time-dependent manner, as expected. However, TRIP treatment induced the time-dependent accumulation of rhenium only in proteins, not in DNA (Figure 1B), which implies that, unlike platinum-based drugs, TRIP induced cancer-selective cytotoxicity by targeting proteins, not DNA.

**TABLE 1:** IC_50_ values of cisplatin, carboplatin, and TRIP in cancer and non-cancer cell lines. Cell lines were treated with each compound for 2 days; then, a colorimetric MTT. metabolic activity assay was conducted to determine cell viability. Data are presented as the mean ± SD (*n* = 3). (a, b) cervical cancer; (c) skin melanoma; (d) embryonic kidney fibroblasts; (e) normal astrocyte.

Compound	IC_50_(uM)				
	HeLa_(a)_	Caski_(b)_	A375P_(c)_	HEK293T_(d)_	C8-D1A_(e)_
Cisplatin	9.20 ± 0.38	7.89 ± 0.26	8.35 ± 0.37	8.33± 0.30	10.19 ± 0.23
Carboplatin	40.48 ± 2.62	22.73 ± 0.20	23.04 ± 0.85	49.64 ± 6.99	23.66 ± 0.21
TRIP	5.30 ± 0.51	5.00 ± 0.86	4.83 ± 0.24	> 100	> 100

### TS-FITGE Identifies Heat Shock Protein 60 as the Target Protein of TRIP

**FIGURE 2:**
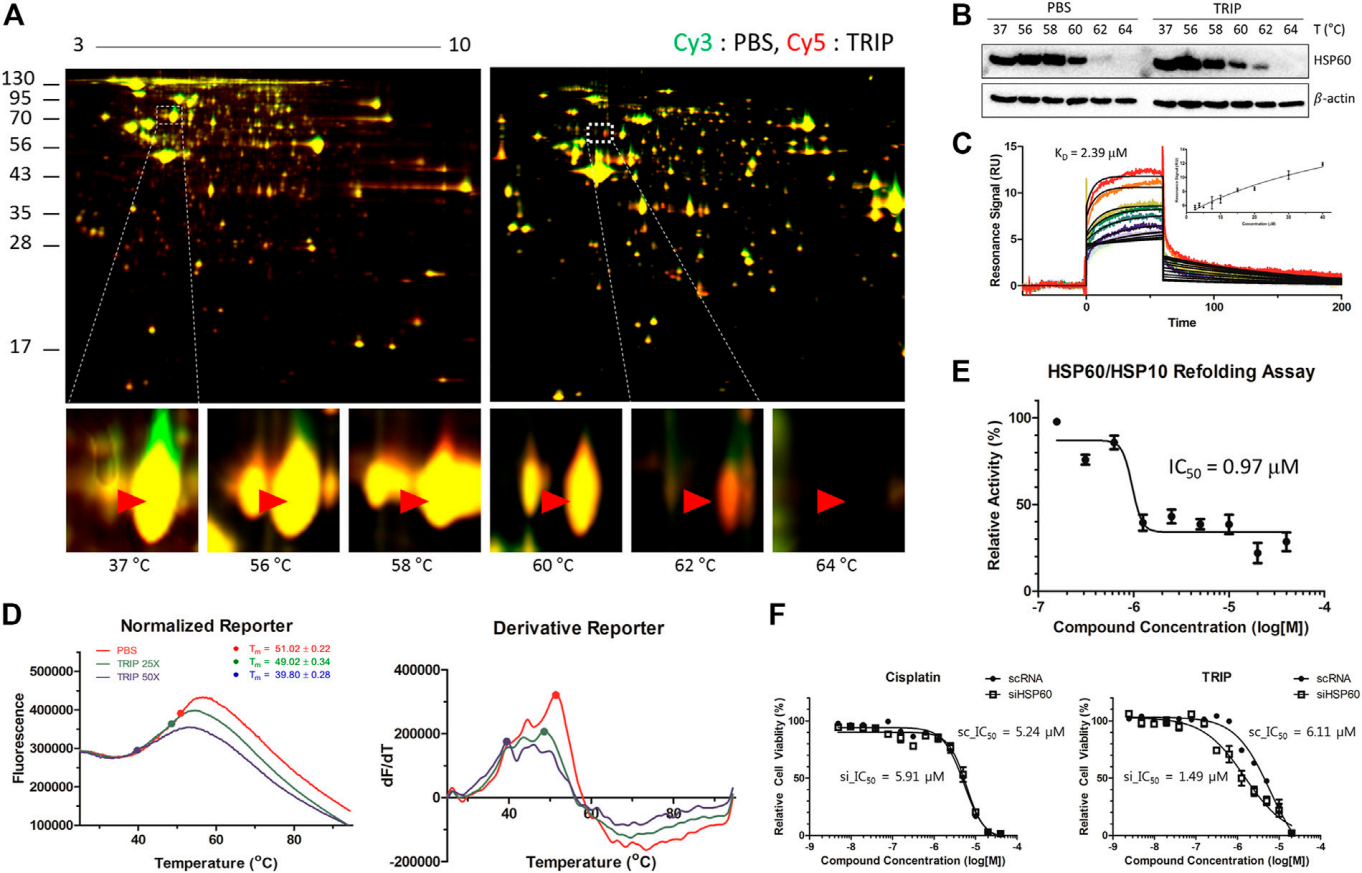
Target identification and target validation of TRIP. **(A)** Representative images of TS-FITGE in HeLa cells. Images of the Cy3 channel (green, PBS-treated) and Cy5 channel (red, 10 μM of TRIP-treated) are overlaid. The area in the white box is magnified. **(B)** Cellular thermal shift assay (CETSA) for specific binding of TRIP to HSP60 in HeLa cells. **(C)** Sensorgrams of surface plasmon resonance (SPR) assay showing the binding kinetics of TRIP (2.5–40 μM) to immobilized HSP60. The dissociation constant (K_D_) value was calculated as the ratio of rate constants (K_d_/K_a_). The inset shows a steady-state response against various concentrations of TRIP (*N* = 3). **(D)** Representative images of melting curves (left) and first derivatives (right) of differential scanning fluorimetry (DSF) analysis demonstrating the binding of TRIP to HSP60. Before heating, 4 μM of purified HSP60 was incubated with 100× Syprox-Orange in the absence or presence of 100 or 200 μM of TRIP for 30 min (*N* = 4). **(E)** Chaperonin functional assay investigating the functional inhibition of TRIP to HSP60. Heated luciferase proteins were incubated with HSP60/HSP10 heterodimer in the absence or presence of TRIP. The luminescence signal was normalized by the vehicle-treated sample (*N* = 5). **(F)** Depletion of HSP60 using siHSP60-2 in HeLa cells showing sensitization of HeLa cells to TRIP, not cisplatin (*N* = 6). Data are presented as the mean ± SD.

Platinum analogs contain various organic ligands to overcome the non-selective cytotoxicity of cisplatin (Go and Adjei, 1999; Cox and Ang, 2009; Riddell and Lippard, 2018). However, since the structural changes in ligands of metal-based complexes can perturb their target engagement (Bruno et al., 2017; Sutton et al., 2019), identifying target proteins without structural changes in metal-based complexes, especially their ligands, would be critical. Therefore, we decided to pursue a label-free target identification for metal-based complexes to overcome specificity. Since TRIP acts on proteins, we conducted a label-free target protein identification that we have developed, namely thermal stability shift-based fluorescence differences in two-dimensional gel electrophoresis (TS-FITGE) (Park et al., 2017). Briefly, HeLa cells were heat-denatured for 3 min at various temperature ranges in the absence or presence of TRIP. Thereafter, the heat-treated cells were lysed, and soluble protein fractions from vehicle- and TRIP-treated conditions were labeled with Cy3 and Cy5 dyes, respectively. Cy3- and Cy5-labeled proteomes from the same temperature point were mixed and analyzed by 2-dimensional gel electrophoresis. Based on color-based image analysis of each gel, we selected a total of nine protein spots with thermal stability shift, including heat-sensitive green and heat-resistant red spots in the presence of TRIP (Supplementary Figure S2, Supplementary Table S1). Among the potential target proteins identified from these spots, we focused on HSP60 (Bukau and Horwich, 1998), a mitochondrial chaperone protein, since we knew that TRIP treatment causes changes in mitochondrial morphology (King et al., 2019). TS-FITGE analysis of the HSP60 spot showed a distinct thermal-stabilized red spot in the 62°C gel (Figure 2A). Cellular thermal shift assay (CETSA) with HSP60-specific antibody further confirmed the thermal stabilization of HSP60 upon TRIP treatment, compared to vehicle, which was consistent with TS-FITGE results (Figure 2B).

To validate the direct binding of TRIP to HSP60, we conducted surface plasmon resonance (SPR) and differential scanning fluorimetry (DSF) analyses. SPR analysis clearly showed a dose-dependent response of the conventional one-on-one binding pattern with a K_D_ value of 2.39 μM (Figure 2C). DSF analysis also showed the melting temperature (T_m_) shift from 51°C to 39.8°C in the presence of TRIP (Figure 2D, Supplementary Figure S3). However, the increased heat sensitivity in DSF was somewhat opposite to TRIP-induced heat resistance of HSP60 observed in TS-FITGE and CETSA experiments. This discrepancy in the thermal stability of HSP60 might be caused by the different states of HSP60 in each experimental setting; TS-FITGE and CETSA were performed in live cells, while DSF was measured with a purified protein. In the cellular system, HSP60 is known to form a heterodimer with heat shock protein 10 (HSP10); however, when purified, HSP60 oligomerizes with itself through protein-protein interactions with a T_m_ value of over 50°C, which can in turn be blocked by HSP60 binders (Shao et al., 2020). When HSP60 disassociates from the oligomer to its monomers, the T_m_ value decreases to 40°C, as observed in DSF analysis in the presence of TRIP. Next, we examined whether the chaperone function of HSP60 can be affected by TRIP. Luciferase protein was denatured by 7-min heat shock at 45°C and incubated with the HSP60/HSP10 complex in the absence or presence of either TRIP or epolactaene *t-*butyl ester (ETB), a known HSP60 inhibitor (Nagumo et al., 2005), for 1.5, 3, and 6 h. Thereafter, the refolded luciferase protein was mixed with luciferin to measure the chaperone activity of HSP60 on the basis of luminescence signals. As shown in Figure 2E, TRIP inhibited the chaperone activity of HSP60 with an IC_50_ value of 0.97 μM (Supplementary Figure S4). Finally, we examined whether the cytotoxic activity of TRIP can be sensitized by siRNA-based knockdown of HSP60 to validate the target specificity of TRIP toward HSP60. Compared to scRNA-treatment, the knockdown of HSP60 significantly sensitized cells to TRIP treatment, although there was no difference in the case of cisplatin (Figure 2F, Supplementary Figure S5). Collectively, our data suggested that TRIP kills cancer cells selectively by inhibiting the chaperone function of HSP60.

### Tricarbonyl Rhenium Isonitrile Polypyridyl Induced Mitochondrial Stress-Mediated p-JNK/AP-1/CHOP Apoptosis

**FIGURE 3:**
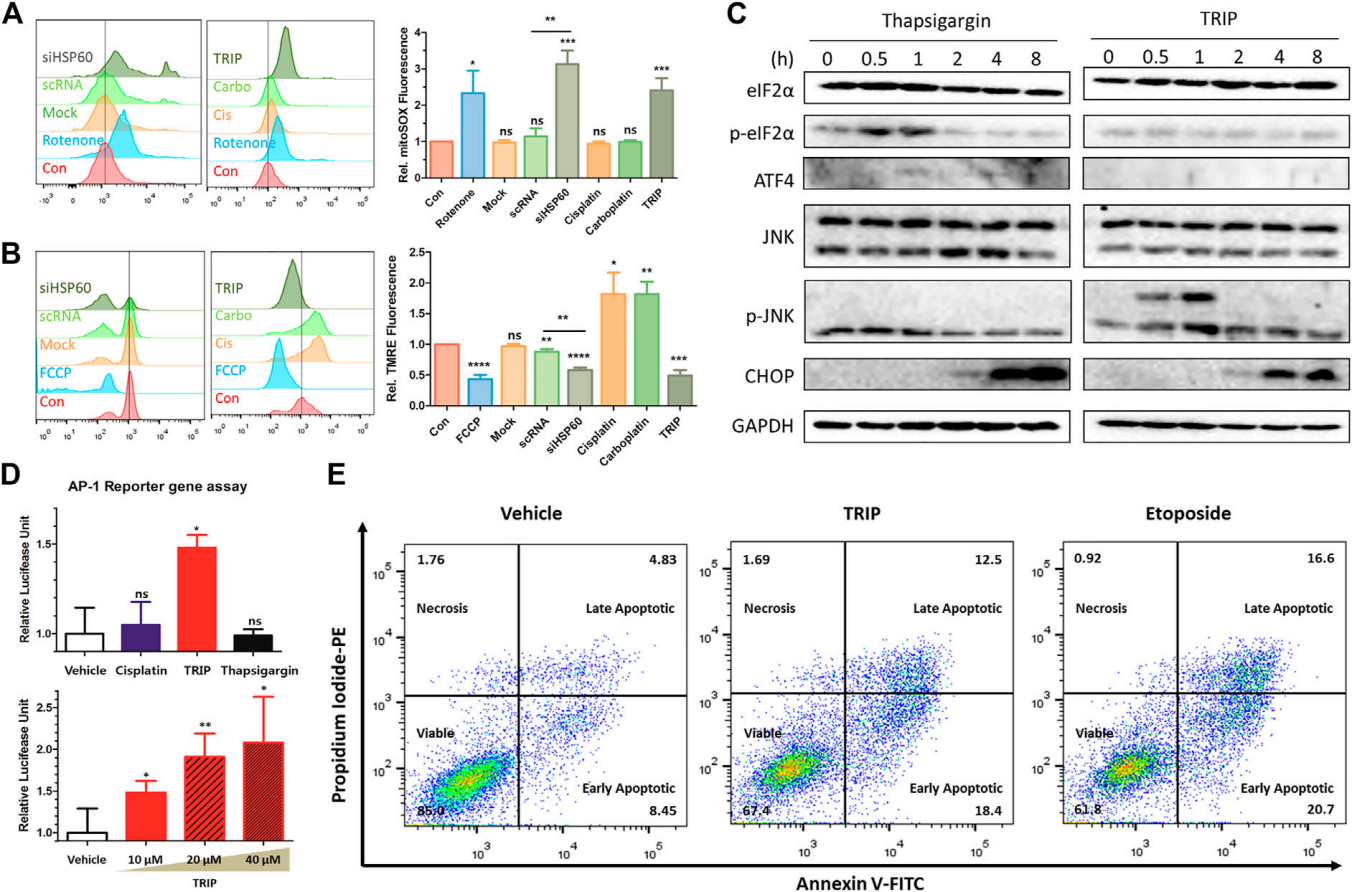
TRIP induced mitochondrial stress-mediated p-JNK/AP-1/CHOP apoptosis. **(A,B)** Flow cytometry data investigating **(A)** an increase of mitochondrial reactive oxygen species (ROS) and **(B)** mitochondrial depolarization upon either TRIP treatment or depletion of HSP60 using siRNA in HeLa cells (*N* = 3). HeLa cells were treated with 10 μM of TRIP for 8 h or transfected with siRNA for 48 h. **(C)** Immunoblot data showing CHOP activation via different signaling pathways in HeLa cells. HeLa cells were treated with either 200 nM of thapsigargin or 10 μM of TRIP at various time points. **(D)** TRIP-mediated AP-1 activation in HeLa cells. 10 μM of each compound was treated for 24 h (upper), and 10, 20, and 40 μM of TRIP were treated for 24 h (lower). Luciferase signal was normalized by the vehicle-treated sample (*N* = 6). Statistical analysis was performed compared to the vehicle group. **(E)** Flow cytometry data showing the alteration in the number of apoptotic cells upon treatment with either TRIP or etoposide in HeLa cells. HeLa cells were treated with either 10 μM of TRIP for 24 h or 50 μM of etoposide for 24 h. Data are presented as the mean ± SD (ns, not significant, *p* > 0.05; *, *p* < 0.05; **, *p* < 0.01; ***, *p* < 0.001; ****, *p* < 0.0001).

TRIP-mediated inhibition of the chaperone function of HSP60 results in the loss of protein homeostasis (proteostasis) and the accumulation of unfolded proteins in mitochondria, with the latter inducing mitochondrial dysfunction and JNK signaling (Mottis et al., 2014; Joy et al., 2021). To quantify mitochondrial stress, we measured the level of reactive oxidative stress (ROS) in mitochondria and their polarization using flow cytometry (Suzuki-Karasaki et al., 2014). Both TRIP treatment and HSP60 knockdown increased mitochondrial ROS levels and induced mitochondrial depolarization (Figures 3A,B). Unfolded protein responses (UPR) in cellular organelles, including endoplasmic reticulum (ER) and mitochondria, induce CHOP-mediated apoptosis in various pathways. For instance, ER-stress inducers (i.e., thapsigargin) activate CHOP *via* p-eIF2α/ATF4 signaling (Rozpedek et al., 2016), and mitochondrial stress inducers (i.e., alkannin) activate CHOP through p-JNK/AP-1 signaling (Aldridge et al., 2007; Horibe and Hoogenraad, 2007; Mottis et al., 2014; Qureshi et al., 2017; Zheng et al., 2020). To validate the specific organelle-derived CHOP activation, we treated HeLa cells with either thapsigargin or TRIP, and observed the subsequent signaling pathways at various time points. Although CHOP activation was observed after 2 h in both conditions, thapsigargin and TRIP activated the p-eIF2α/ATF4 and p-JNK2 signaling pathways, respectively, within 1 h (Figure 3C). AP-1 reporter gene assay confirmed that only TRIP activated p-JNK/AP-1 signaling as a mitochondrial stress inducer, unlike cisplatin or thapsigargin (Figure 3D). To validate p-JNK/AP-1/CHOP-mediated apoptosis, we treated HeLa cells with TRIP or etoposide―an apoptosis inducer by modulating the function of topoisomerase II. Flow cytometry confirmed that both TRIP and etoposide kill HeLa cells via apoptosis, not necrosis (Figure 3E, Supplementary Figure S6). Furthermore, the time- and dose-dependent TRIP treatment significantly increased the number of cells that entered early and late apoptosis compared to vehicle treatment (Supplementary Figure S7).

### Cancer Cells Utilized Mitochondrial Fission to Reduce Mitochondrial Stress

**FIGURE 4:**
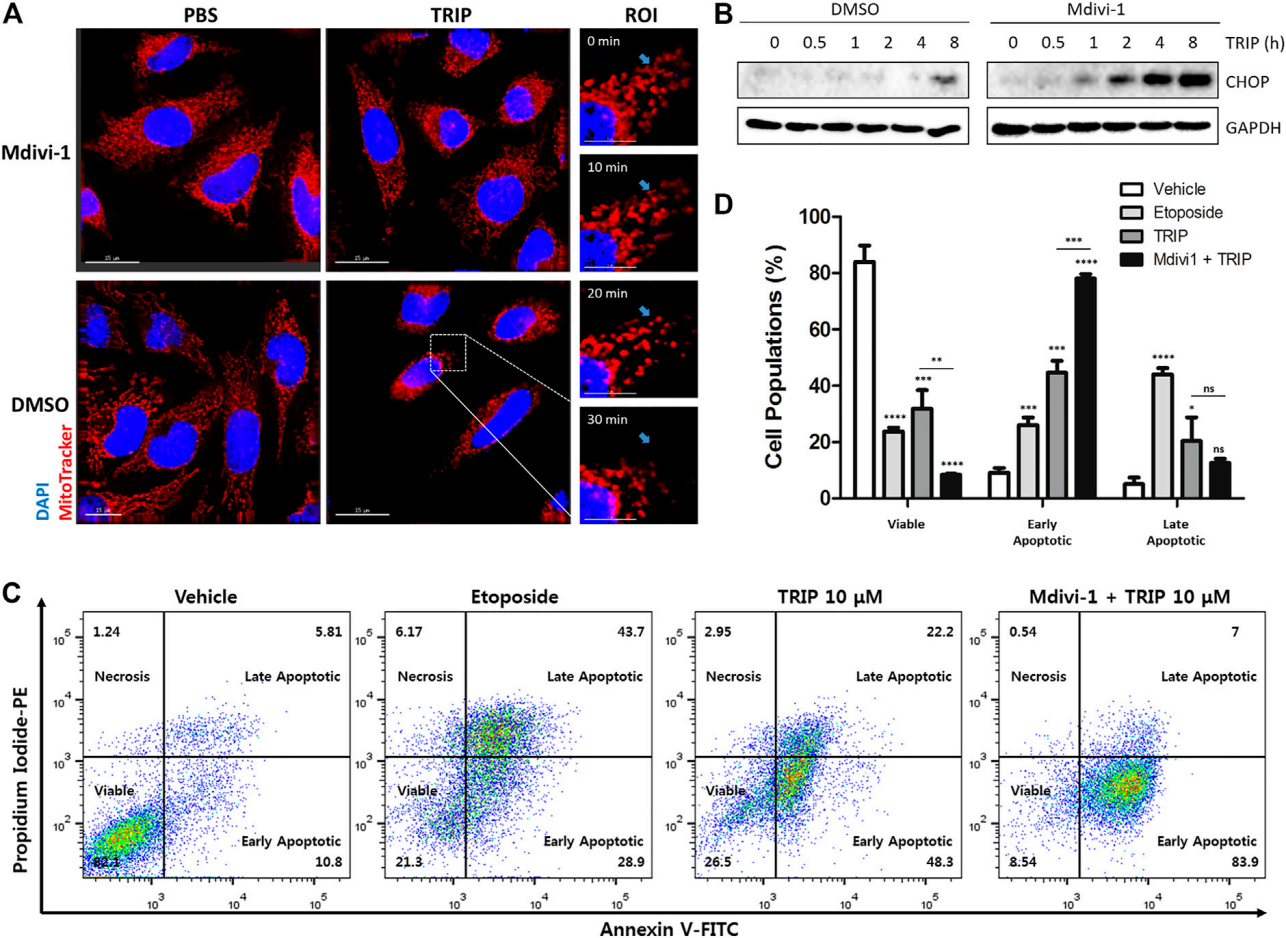
Cancer cells utilize mitochondrial fission to reduce mitochondrial stress. **(A)** Immunofluorescence images showing mitochondrial fission induced by TRIP and perturbed by Mdivi-1 in HeLa cells. **(B)** Immunoblot data in HeLa cells demonstrating CHOP activation enhanced by 50 μM of Mdivi-1 co-treatment with TRIP. **(C)** Flow cytometry data showing CHOP-mediated apoptosis reinforced by 50 μM of Mdivi-1 co-treatment with TRIP in HeLa cells. **(D)** Quantitative data pertaining to **(C)** (*N* = 3). Data are presented as the mean ± SD (ns, not significant, *p* > 0.05; *, *p* < 0.05; **, *p* < 0.01; ***, *p* < 0.001; ****, *p* < 0.0001). R.O.I., region of interest.

TRIP treatment induces mitochondrial fission that can be blocked by Mdivi-1, an inhibitor for dynamin-related protein 1 (Drp1)―a crucial mediator of mitochondrial fission (King et al., 2019). As shown in Figure 4A, our immunofluorescence images suggested that TRIP treatment induces mitochondrial fission in a time-dependent manner, which in turn could be blocked by Mdivi-1 treatment. Therefore, to examine whether TRIP-induced mitochondrial fission triggers CHOP-mediated apoptosis or relieves mitochondrial stress, we treated HeLa cells with TRIP in the absence or presence of Mdivi-1 at various time points. Interestingly, Mdivi-1 accelerated TRIP-induced CHOP activation, but Mdivi-1 did not boost thapsigargin-induced CHOP activation (Figure 4B, Supplementary Figure S8), indicating that cancer cells utilize mitochondrial fission to reduce mitochondrial stress. To validate this observation, we conducted flow cytometry analysis. Compared to the TRIP-only treatment, the co-treatment of TRIP with Mdivi-1 increased early apoptotic cells by approximately two-fold without significant changes in the late apoptotic cells (Figures 4C,D), confirming the acceleration of TRIP-mediated apoptotic cell death by inhibiting mitochondrial fission. When the TRIP-treatment time was extended to 48 h, Mdivi-1 significantly increased the late apoptotic cell death (Supplementary Figure S9). However, Mdivi-1 did not influence the portion of late apoptotic cells in the case of etoposide-mediated cell death via DNA double-strand break, which is orthogonal to TRIP-mediated apoptotic cell death via the accumulation of unfolded proteins in mitochondria (Supplementary Figure S10).

## Conclusion

**FIGURE 5:**
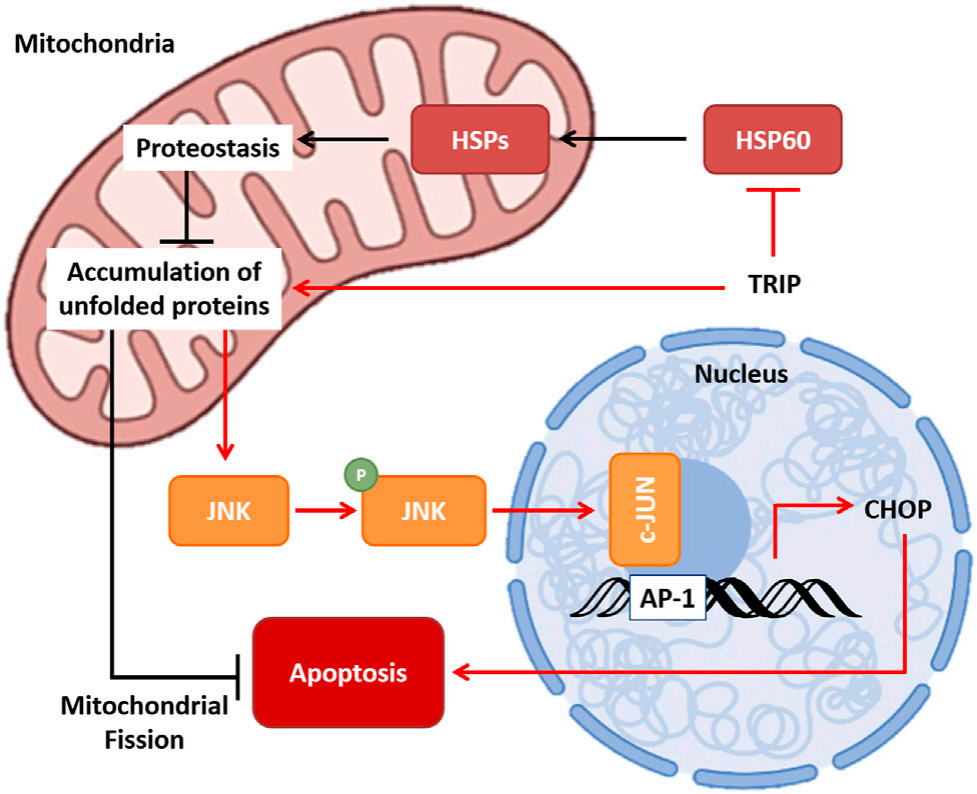
Molecular mechanism of TRIP. TRIP disrupts proteostasis, which leads to the accumulation of unfolded proteins in mitochondria via inhibiting the chaperone function of HSP60. Mitochondrial unfolded protein response (mtUPR) activates p-JNK/AP-1/CHOP-mediated apoptosis, and cancer cells utilize mitochondrial fission to prevent apoptosis.

In this study, based on ICP-MS analysis, we revealed that TRIP targets protein, not DNA. Furthermore, we identified HSP60 as the target protein of TRIP by a label-free target identification method, TS-FITGE. We also confirmed that TRIP directly binds to HSP60 and inhibits its chaperone function, eventually leading to cancer-specific cell death. Inhibition of the chaperone function of HSP60 disrupts cellular proteostasis and causes the accumulation of unfolded proteins in mitochondria. The resulting mtUPR activates the p-JNK2/AP-1/CHOP-mediated apoptotic pathway, exacerbated by inhibiting mitochondrial fission (Figure 5). Collectively, our study revealed how a rhenium-based compound could selectively induce apoptosis of cancer cells at the molecular level and demonstrated label-free target identification as a potential and indispensable tool for unveiling the molecular mechanism of metal-based drugs.

## Data Availability

The original contributions presented in the study are included in the article/Supplementary Material, further inquiries can be directed to the corresponding author.
