# Supplementary material for: Label-Free Target Identification Reveals the Anticancer Mechanism of a Rhenium Isonitrile Complex
Source: Front Chem. 2022 Mar 14;10:850638. doi: 10.3389/fchem.2022.850638 (PMC8964423; doi:10.3389/fchem.2022.850638)

## Slide 1
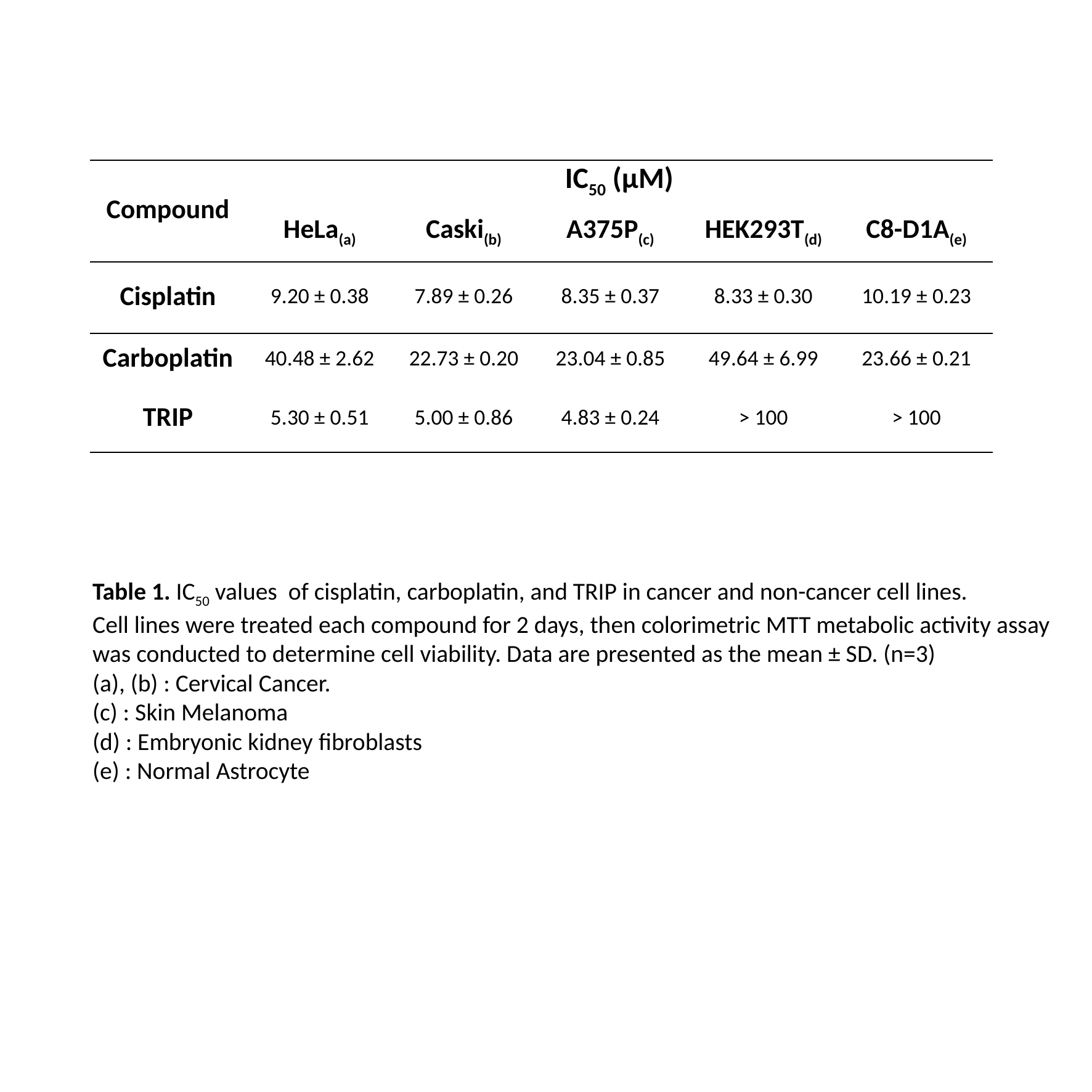

| Compound | IC50 (μM) | | | | |
| --- | --- | --- | --- | --- | --- |
| | HeLa(a) | Caski(b) | A375P(c) | HEK293T(d) | C8-D1A(e) |
| Cisplatin | 9.20 ± 0.38 | 7.89 ± 0.26 | 8.35 ± 0.37 | 8.33 ± 0.30 | 10.19 ± 0.23 |
| Carboplatin | 40.48 ± 2.62 | 22.73 ± 0.20 | 23.04 ± 0.85 | 49.64 ± 6.99 | 23.66 ± 0.21 |
| TRIP | 5.30 ± 0.51 | 5.00 ± 0.86 | 4.83 ± 0.24 | > 100 | > 100 |
Table 1. IC50 values of cisplatin, carboplatin, and TRIP in cancer and non-cancer cell lines.
Cell lines were treated each compound for 2 days, then colorimetric MTT metabolic activity assay was conducted to determine cell viability. Data are presented as the mean ± SD. (n=3)
(a), (b) : Cervical Cancer.
(c) : Skin Melanoma
(d) : Embryonic kidney fibroblasts
(e) : Normal Astrocyte

## Slide 2
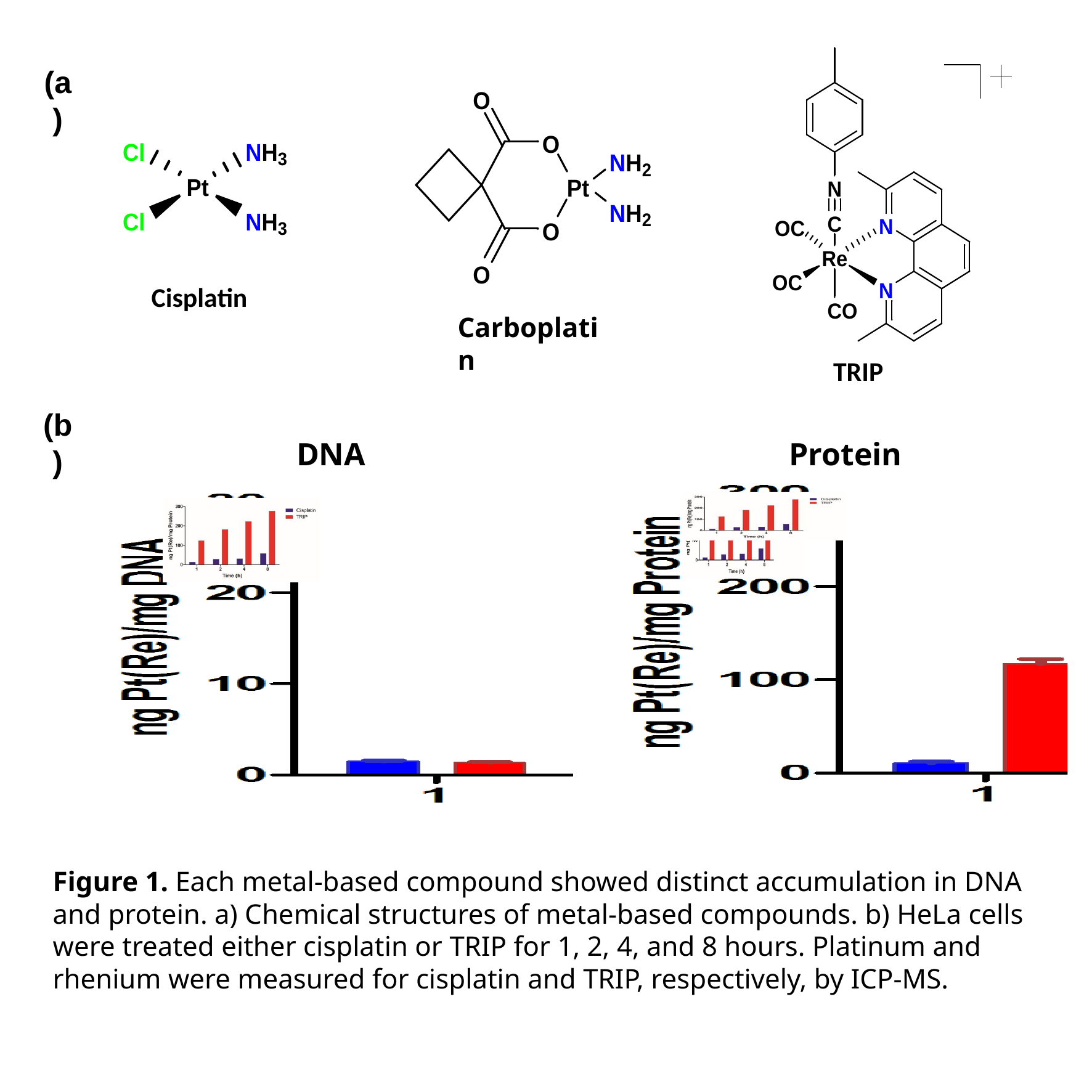

TRIP
(a)
Carboplatin
Cisplatin
(b)
DNA
Protein
Figure 1. Each metal-based compound showed distinct accumulation in DNA and protein. a) Chemical structures of metal-based compounds. b) HeLa cells were treated either cisplatin or TRIP for 1, 2, 4, and 8 hours. Platinum and rhenium were measured for cisplatin and TRIP, respectively, by ICP-MS.

## Slide 3
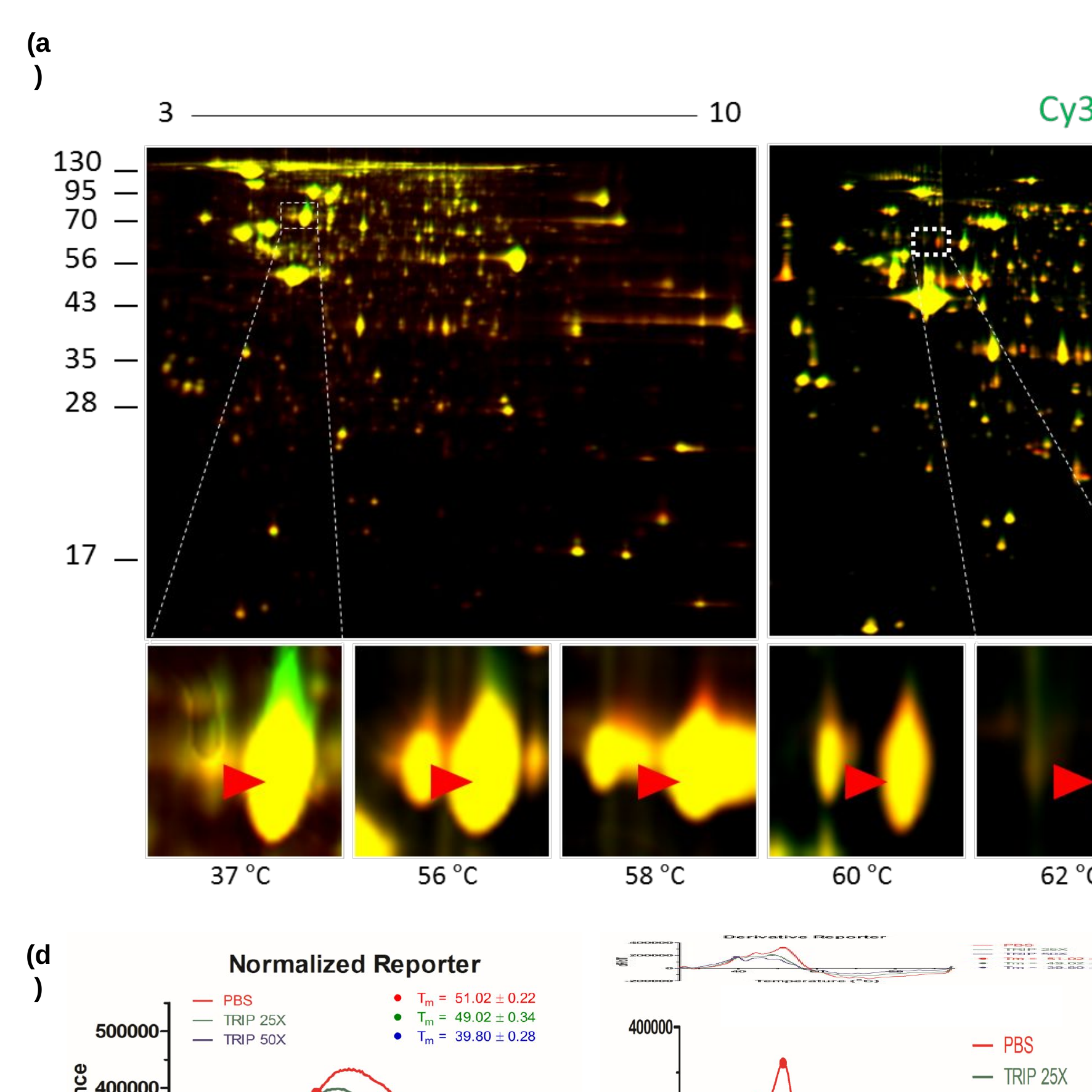

(a)
(b)
(c)
(e)
(d)
(f)
Figure 2. Target identification and target validation of TRIP. a) Representative images of TS-FITGE in HeLa cells. Images of the Cy3 channel (green, PBS-treated) and Cy5 channel (Red, 10 μM of TRIP-treated) are overlaid. The area in the white box is magnified. b) CETSA for specific binding of TRIP to HSP60 in HeLa cells. c) Sensorgrams of surface plasmon resonance (SPR) assay showing the binding kinetics of TRIP (2.5 to 40 μM) to immobilized HSP60. The dissociation constant (KD) value was calculated as the ratio of rate constants (Kd/Ka). The inset shows a steady-state response against various concentrations of TRIP (n=3). d) Representative image of melting curves (upper) and first derivatives (lower) of differential scanning fluorimetry (DSF) analysis demonstrating the binding of TRIP to HSP60. Before heating, 4 μM of purified HSP60 was incubated with 100X syprox-orange in the absence or presence of 100 or 200 μM of TRIP for 30 min (n=4). e) Chaperonin functional assay investigating functional inhibition of TRIP to HSP60. Heated luciferase proteins were incubated with HSP60/HSP10 heterodimer in the absence or presence of TRIP. Luminescence signal was normalized by the vehicle-treated sample (n=5). f) Depletion of HSP60 using siHSP60-2 in HeLa cells showing sensitization of HeLa cells to TRIP, not cisplatin (n=6). Data are presented as the mean ± SD.

## Slide 4
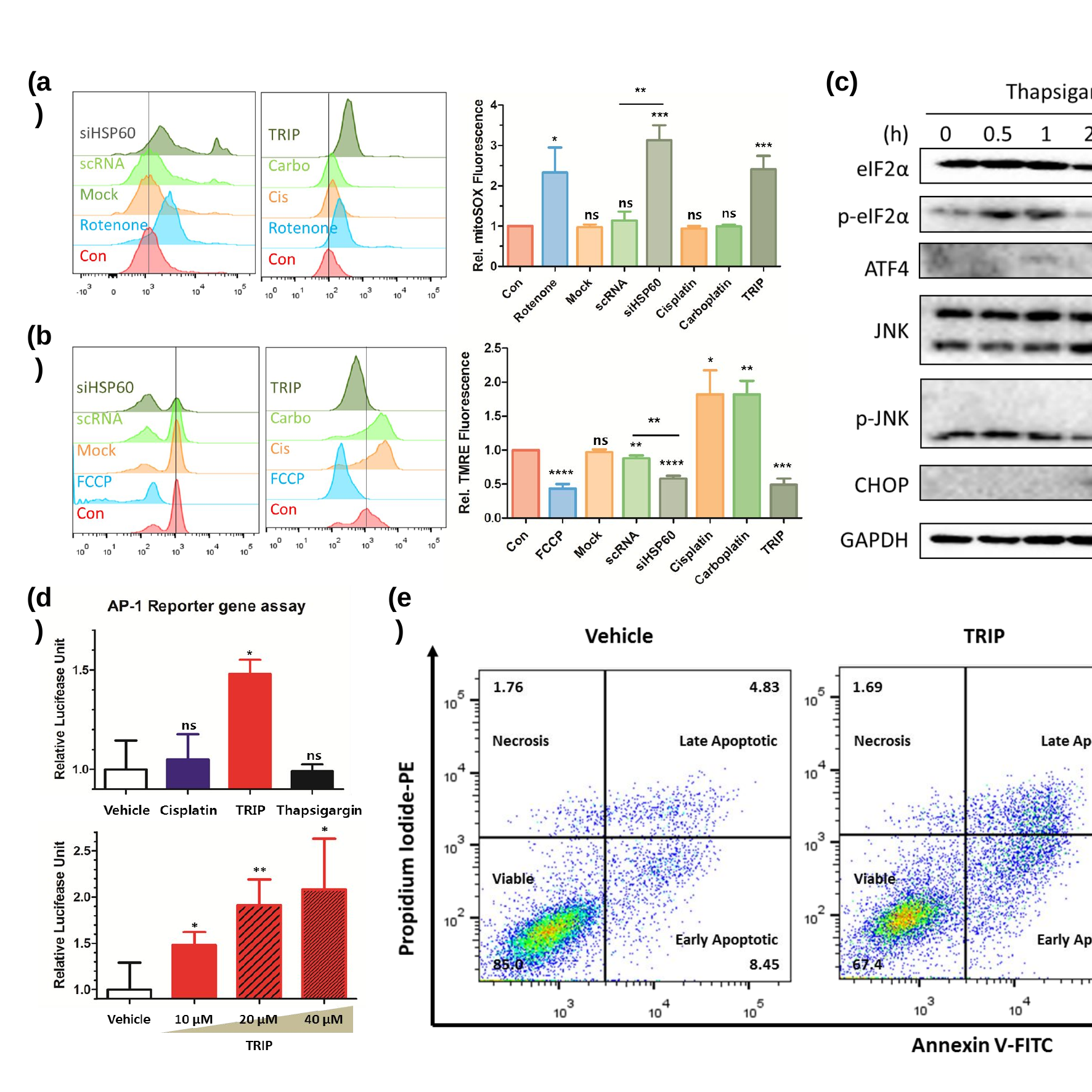

(a)
(c)
(b)
(d)
(e)
Figure 3. TRIP induced mitochondrial stress-mediated p-JNK/AP-1/CHOP apoptosis. a-b) Flow cytometry data investigating (a) an increase of mitochondrial ROS and (b) mitochondrial depolarization upon either TRIP treatment or depletion of HSP60 using siRNA in HeLa cells (n=3). HeLa cells were treated with 10 μM of TRIP for 8 h or transfected with siRNA for 48 h. c) Immunoblot data showing CHOP activation via different signaling pathways in HeLa cells. HeLa cells were treated with either 200 nM of thapsigargin or 10 μM of TRIP at various time points. d) TRIP-mediated AP-1 activation in HeLa cells. 10 μM of each compounds was treated for 24 h (upper) and 10, 20, and 40 μM of TRIP was treated for 24 h (lower). Luciferase signal was normalized by the vehicle-treated sample (n=6). e) Flow cytometry data showing the alteration in the number of apoptotic cells upon treatment of either TRIP or etoposide in HeLa cells. HeLa cells were treated with either 10 μM of TRIP for 24 h or 50 μM of etoposide for 24 h. Data are presented as the mean ± SD (ns, not significant, p >0.05; *, p < 0.05; **, p < 0.01; ***, p < 0.001; ****, p < 0.0001).

## Slide 5
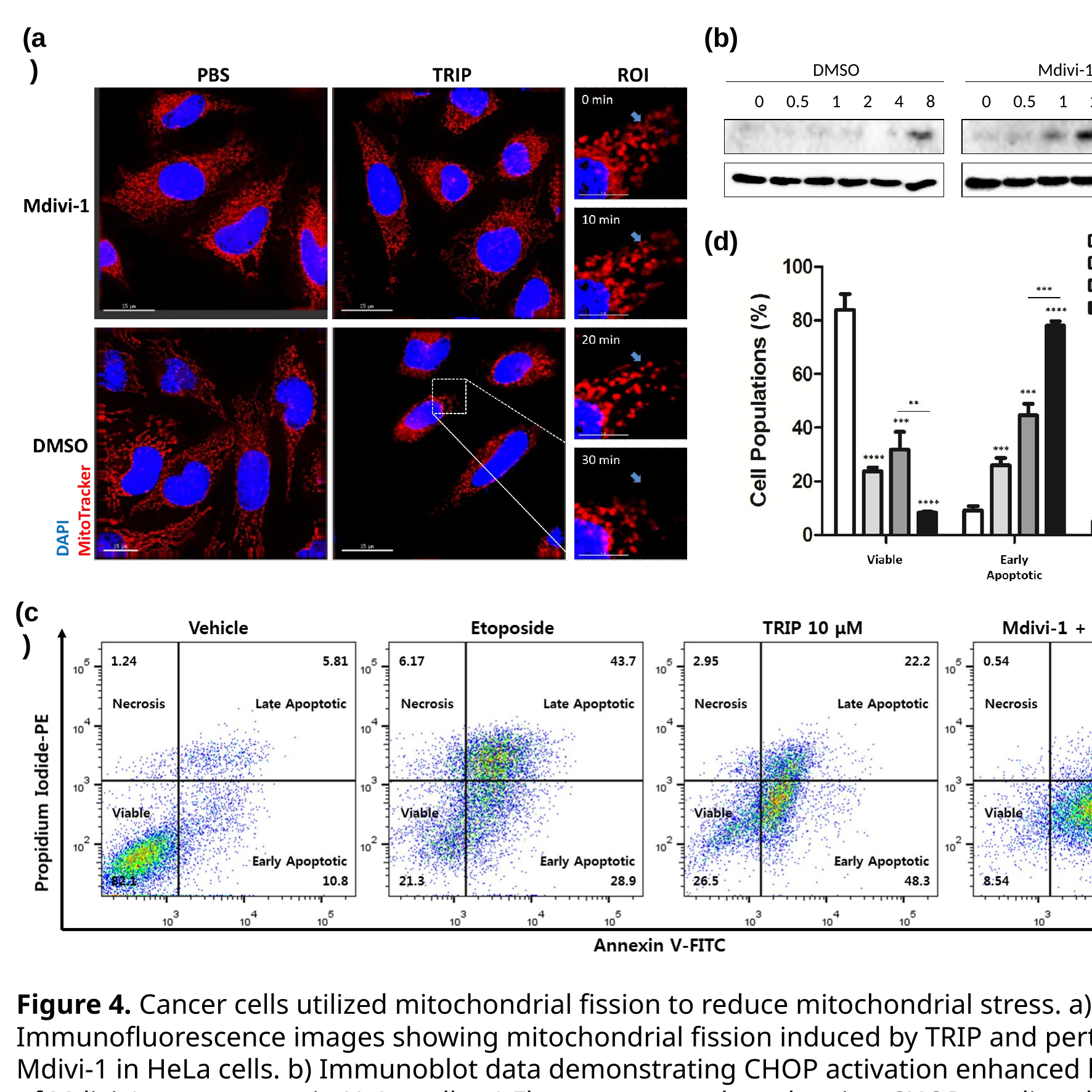

(a)
(b)
DMSO
Mdivi-1
0
0.5
1
2
4
8
0
0.5
1
2
4
8
TRIP (h)
CHOP
GAPDH
(d)
(c)
Figure 4. Cancer cells utilized mitochondrial fission to reduce mitochondrial stress. a) Immunofluorescence images showing mitochondrial fission induced by TRIP and perturbed by Mdivi-1 in HeLa cells. b) Immunoblot data demonstrating CHOP activation enhanced by 50 μM of Mdivi-1 cotreatment in HeLa cells. c) Flow cytometry data showing CHOP-mediated apoptosis reinforced by 50 μM of Mdivi-1 cotreatment in HeLa cells. d) Quantitative data pertaining to (c). Data are presented as the mean ± SD (ns, not significant, p >0.05; *, p < 0.05; **, p < 0.01; ***, p < 0.001; ****, p < 0.0001). ROI, region of interest.

## Slide 6
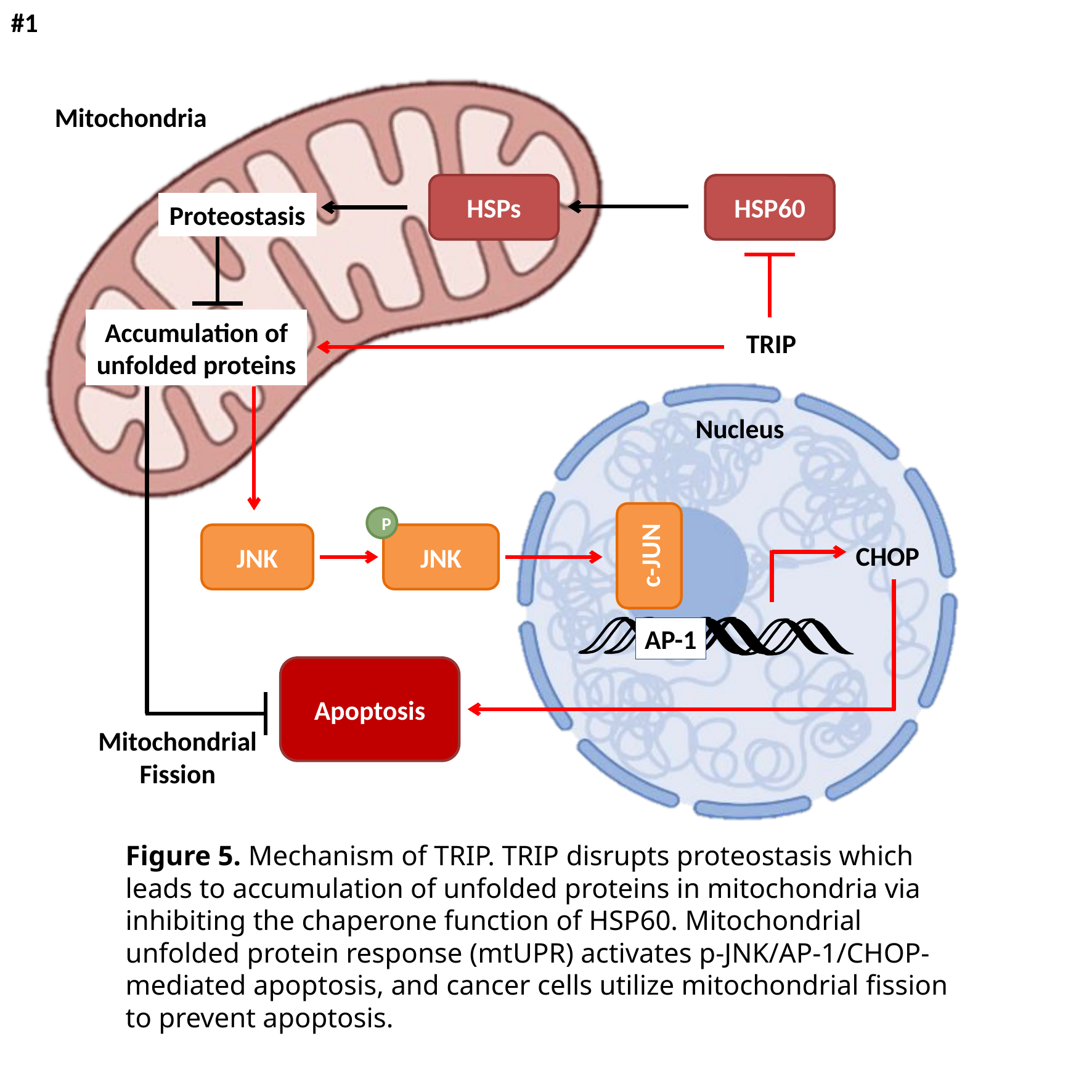

#1
Mitochondria
HSPs
HSP60
Proteostasis
Accumulation of
unfolded proteins
TRIP
Nucleus
P
c-JUN
JNK
JNK
CHOP
AP-1
Apoptosis
Mitochondrial
Fission
Figure 5. Mechanism of TRIP. TRIP disrupts proteostasis which leads to accumulation of unfolded proteins in mitochondria via inhibiting the chaperone function of HSP60. Mitochondrial unfolded protein response (mtUPR) activates p-JNK/AP-1/CHOP-mediated apoptosis, and cancer cells utilize mitochondrial fission to prevent apoptosis.

## Slide 7
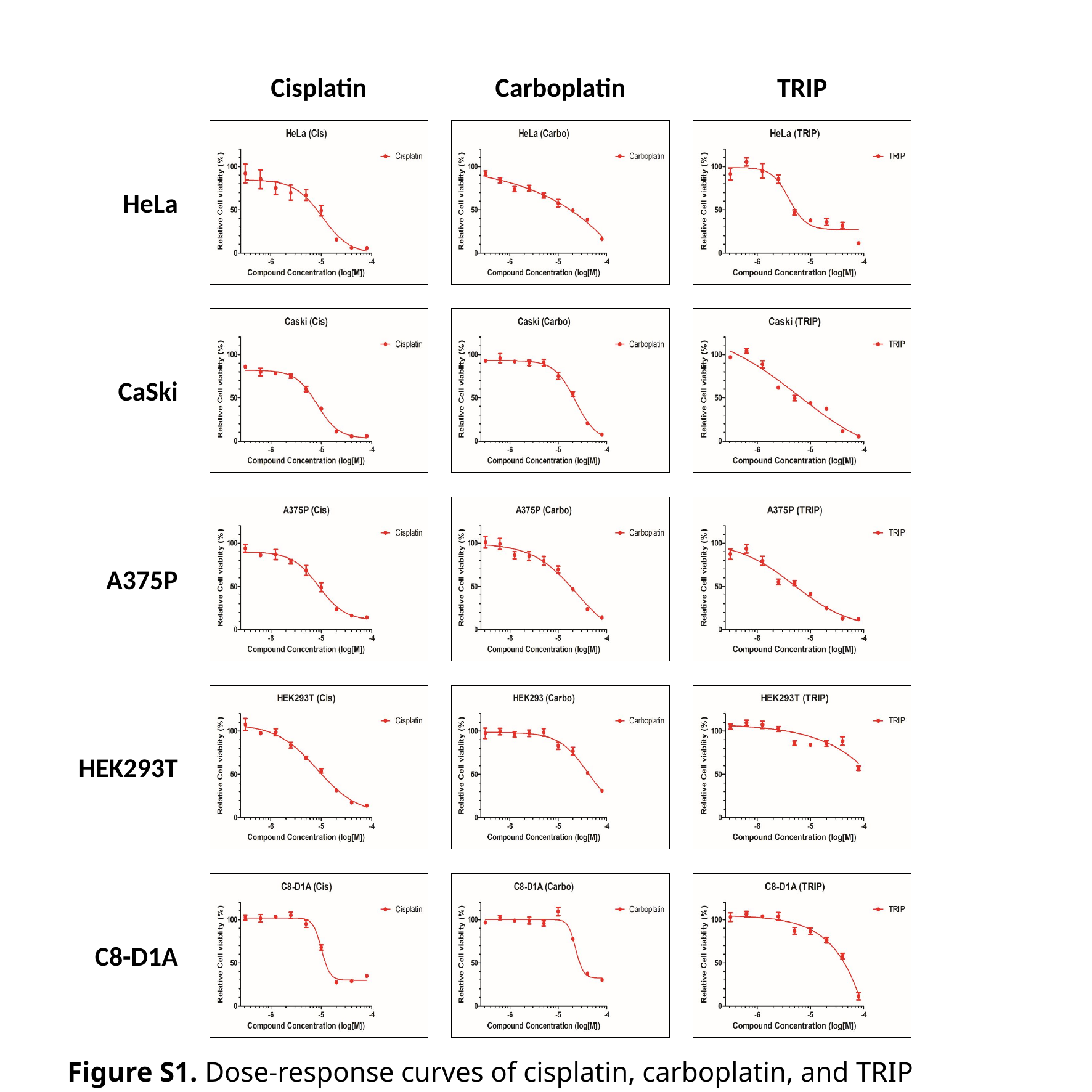

Cisplatin
Carboplatin
TRIP
HeLa
CaSki
A375P
HEK293T
C8-D1A
Figure S1. Dose-response curves of cisplatin, carboplatin, and TRIP in various cell lines (n=6). These data are summarized in Table 1. Data are presented as the mean ± SD.

## Slide 8
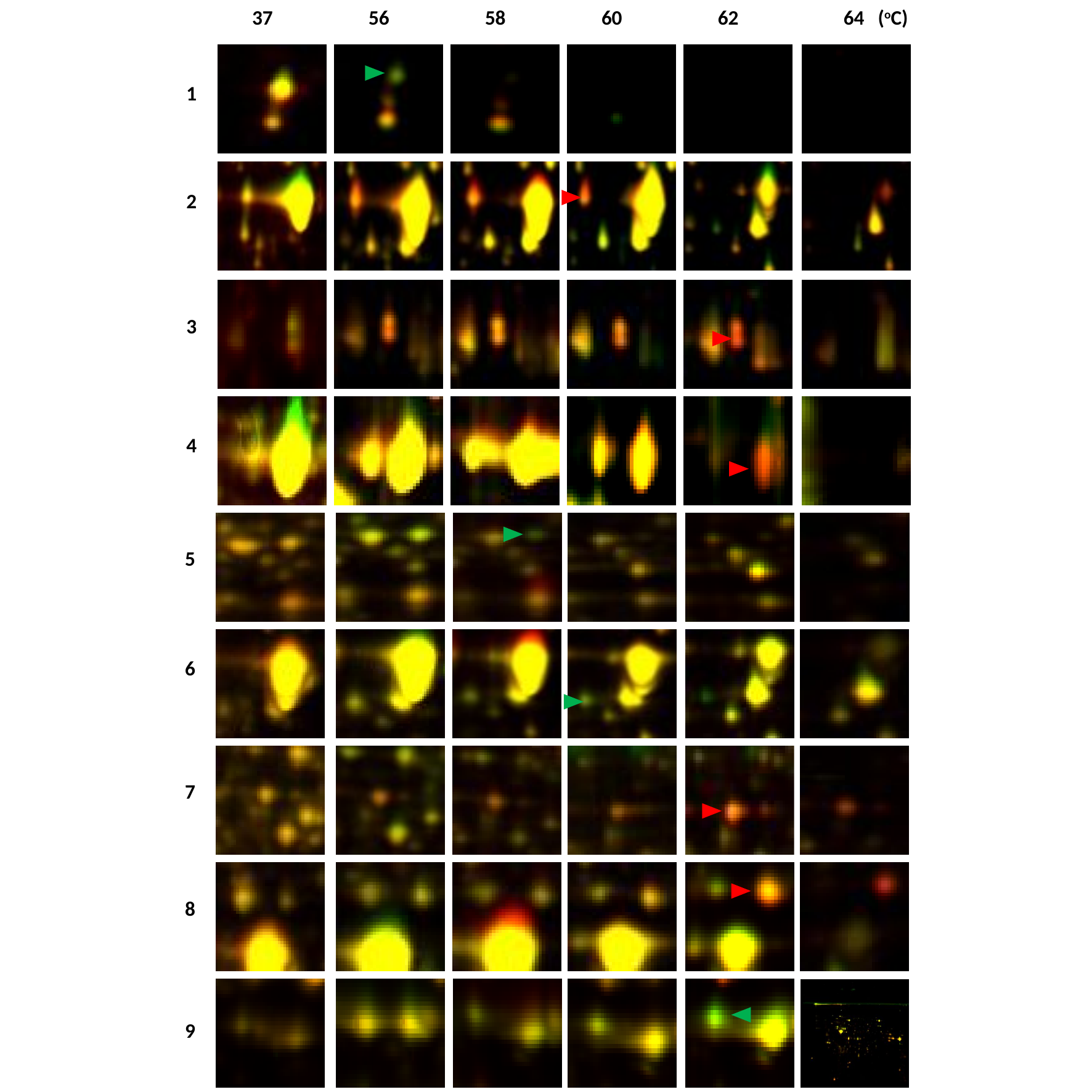

37
56
58
60
62
64 (oC)
1
2
3
4
5
6
7
8
9
Figure S2. Observed nine spots including heat-sensitive green spots and heat-resistant red spots in TS-FITGE.

## Slide 9
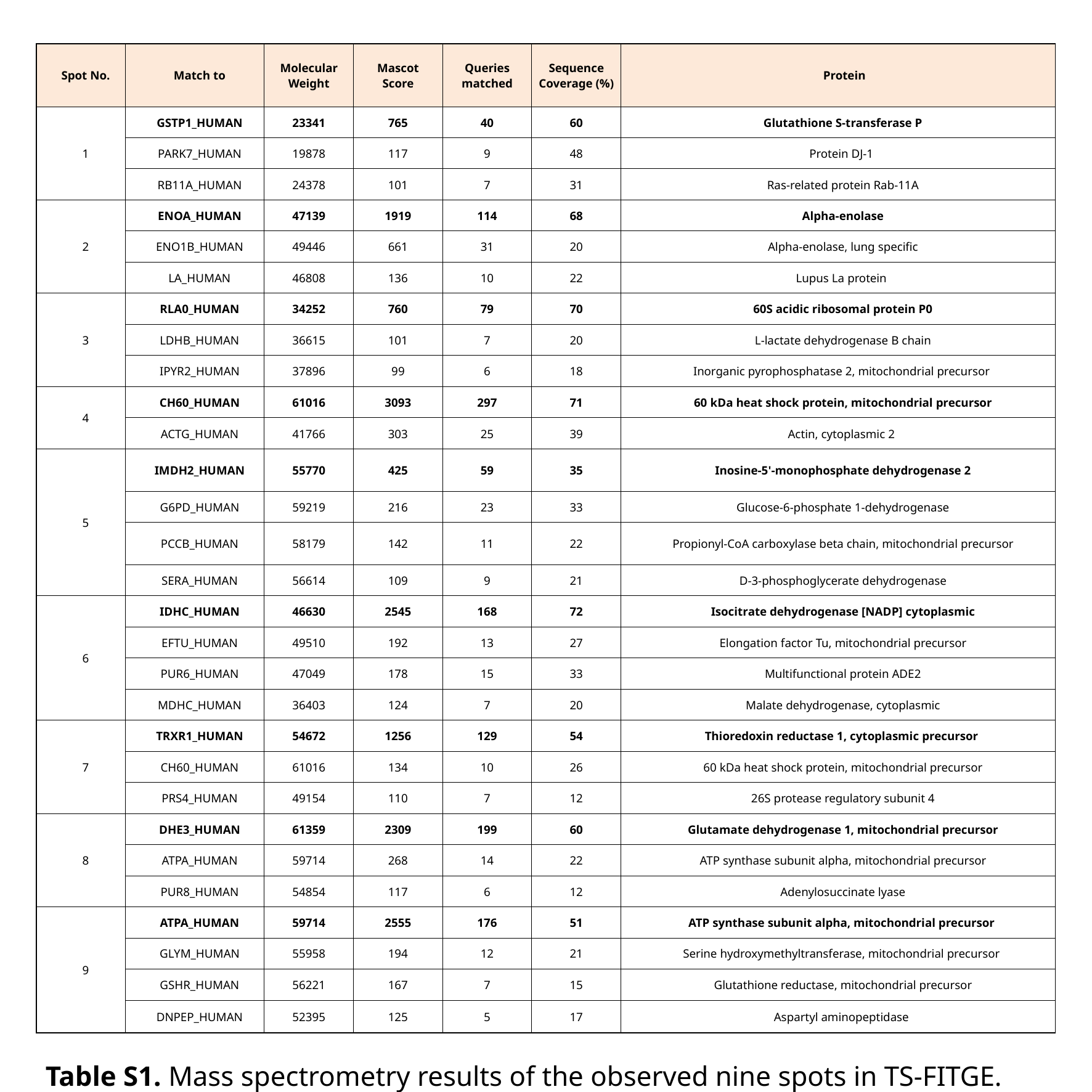

| Spot No. | Match to | Molecular Weight | Mascot Score | Queries matched | Sequence Coverage (%) | Protein |
| --- | --- | --- | --- | --- | --- | --- |
| 1 | GSTP1\_HUMAN | 23341 | 765 | 40 | 60 | Glutathione S-transferase P |
| | PARK7\_HUMAN | 19878 | 117 | 9 | 48 | Protein DJ-1 |
| | RB11A\_HUMAN | 24378 | 101 | 7 | 31 | Ras-related protein Rab-11A |
| 2 | ENOA\_HUMAN | 47139 | 1919 | 114 | 68 | Alpha-enolase |
| | ENO1B\_HUMAN | 49446 | 661 | 31 | 20 | Alpha-enolase, lung specific |
| | LA\_HUMAN | 46808 | 136 | 10 | 22 | Lupus La protein |
| 3 | RLA0\_HUMAN | 34252 | 760 | 79 | 70 | 60S acidic ribosomal protein P0 |
| | LDHB\_HUMAN | 36615 | 101 | 7 | 20 | L-lactate dehydrogenase B chain |
| | IPYR2\_HUMAN | 37896 | 99 | 6 | 18 | Inorganic pyrophosphatase 2, mitochondrial precursor |
| 4 | CH60\_HUMAN | 61016 | 3093 | 297 | 71 | 60 kDa heat shock protein, mitochondrial precursor |
| | ACTG\_HUMAN | 41766 | 303 | 25 | 39 | Actin, cytoplasmic 2 |
| 5 | IMDH2\_HUMAN | 55770 | 425 | 59 | 35 | Inosine-5'-monophosphate dehydrogenase 2 |
| | G6PD\_HUMAN | 59219 | 216 | 23 | 33 | Glucose-6-phosphate 1-dehydrogenase |
| | PCCB\_HUMAN | 58179 | 142 | 11 | 22 | Propionyl-CoA carboxylase beta chain, mitochondrial precursor |
| | SERA\_HUMAN | 56614 | 109 | 9 | 21 | D-3-phosphoglycerate dehydrogenase |
| 6 | IDHC\_HUMAN | 46630 | 2545 | 168 | 72 | Isocitrate dehydrogenase [NADP] cytoplasmic |
| | EFTU\_HUMAN | 49510 | 192 | 13 | 27 | Elongation factor Tu, mitochondrial precursor |
| | PUR6\_HUMAN | 47049 | 178 | 15 | 33 | Multifunctional protein ADE2 |
| | MDHC\_HUMAN | 36403 | 124 | 7 | 20 | Malate dehydrogenase, cytoplasmic |
| 7 | TRXR1\_HUMAN | 54672 | 1256 | 129 | 54 | Thioredoxin reductase 1, cytoplasmic precursor |
| | CH60\_HUMAN | 61016 | 134 | 10 | 26 | 60 kDa heat shock protein, mitochondrial precursor |
| | PRS4\_HUMAN | 49154 | 110 | 7 | 12 | 26S protease regulatory subunit 4 |
| 8 | DHE3\_HUMAN | 61359 | 2309 | 199 | 60 | Glutamate dehydrogenase 1, mitochondrial precursor |
| | ATPA\_HUMAN | 59714 | 268 | 14 | 22 | ATP synthase subunit alpha, mitochondrial precursor |
| | PUR8\_HUMAN | 54854 | 117 | 6 | 12 | Adenylosuccinate lyase |
| 9 | ATPA\_HUMAN | 59714 | 2555 | 176 | 51 | ATP synthase subunit alpha, mitochondrial precursor |
| | GLYM\_HUMAN | 55958 | 194 | 12 | 21 | Serine hydroxymethyltransferase, mitochondrial precursor |
| | GSHR\_HUMAN | 56221 | 167 | 7 | 15 | Glutathione reductase, mitochondrial precursor |
| | DNPEP\_HUMAN | 52395 | 125 | 5 | 17 | Aspartyl aminopeptidase |
Table S1. Mass spectrometry results of the observed nine spots in TS-FITGE.

## Slide 10
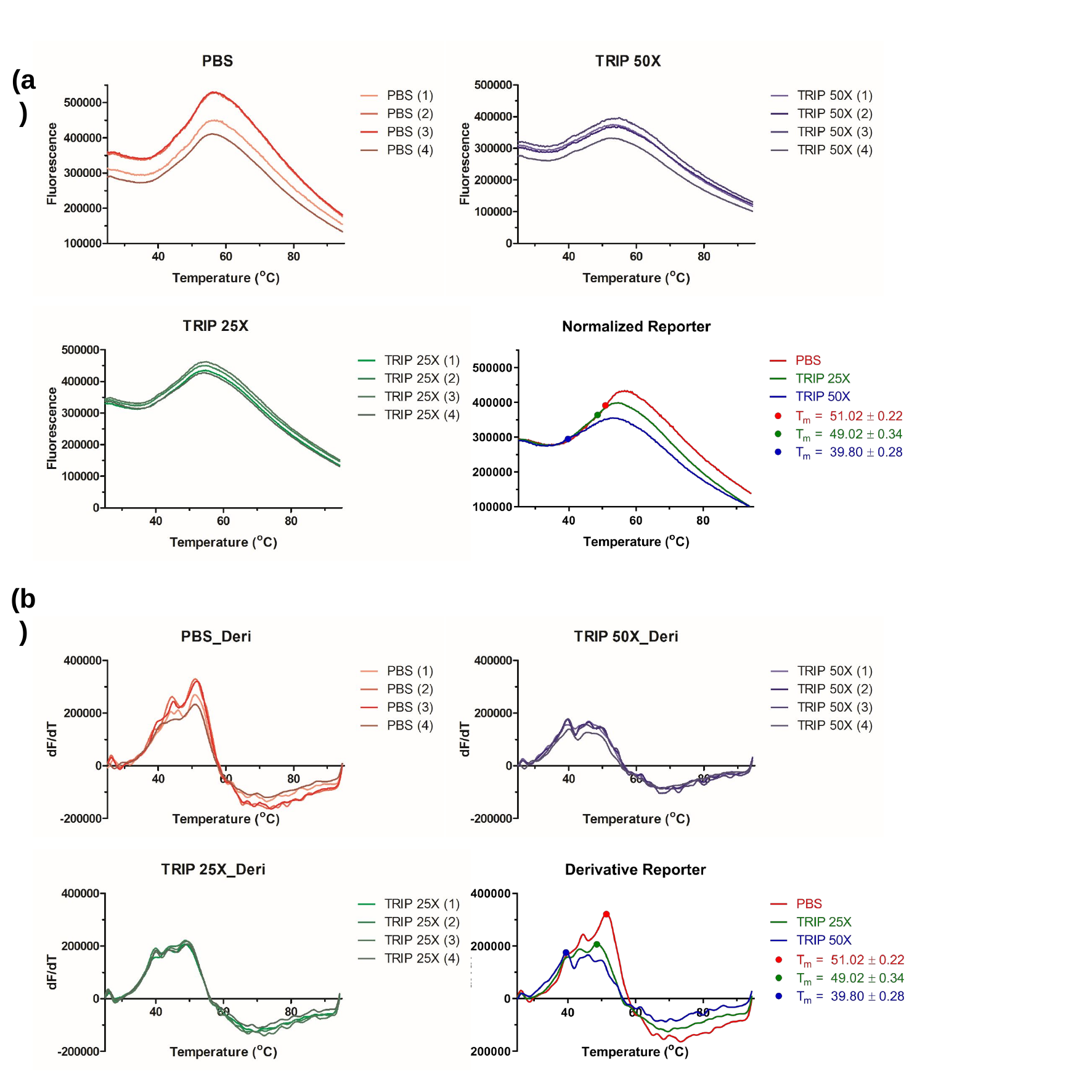

(a)
(b)
Figure S3. Full data of melting curves (a) and first derivatives (b) of differential scanning fluorimetry (DSF) analysis (n=4).

## Slide 11
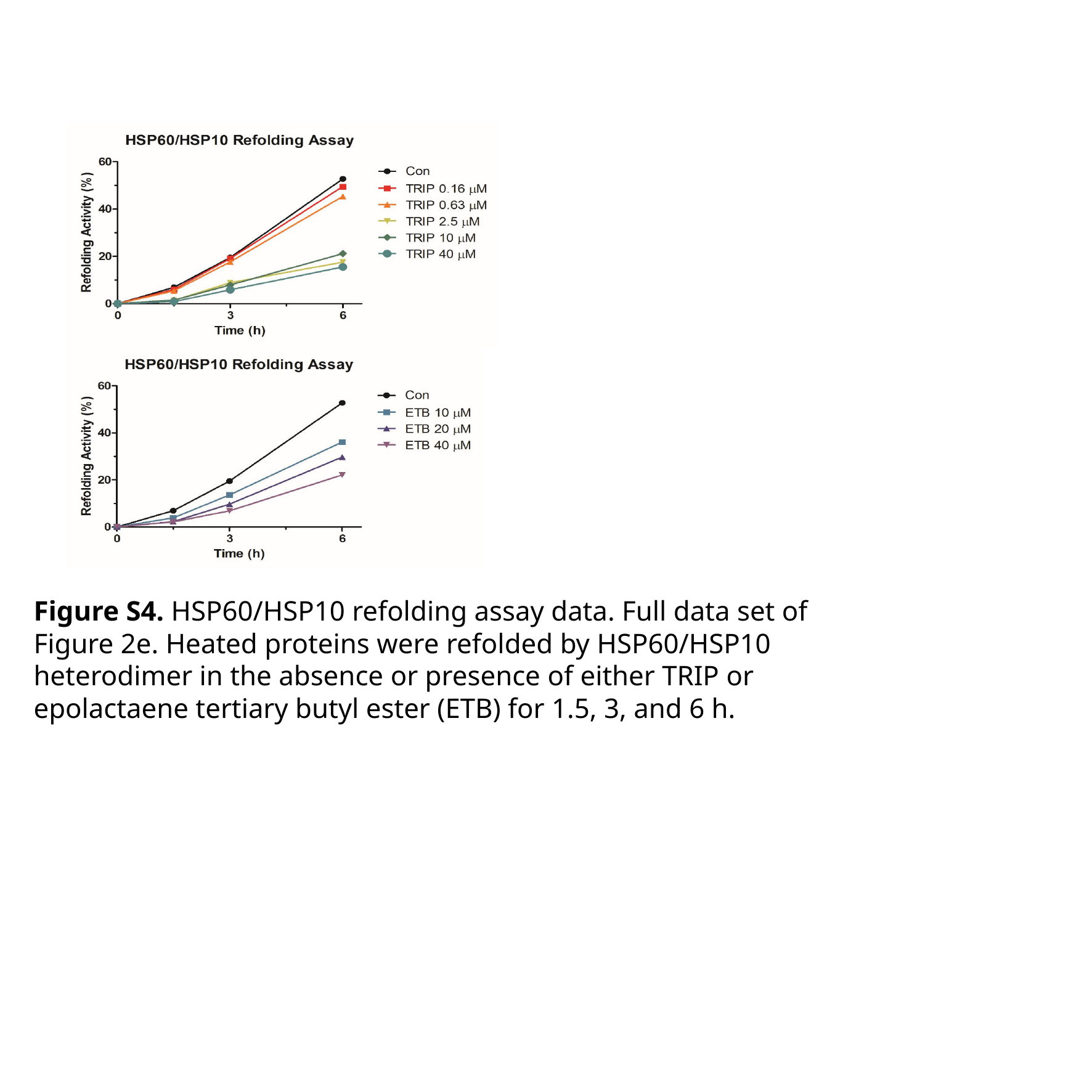

Figure S4. HSP60/HSP10 refolding assay data. Full data set of Figure 2e. Heated proteins were refolded by HSP60/HSP10 heterodimer in the absence or presence of either TRIP or epolactaene tertiary butyl ester (ETB) for 1.5, 3, and 6 h.

## Slide 12
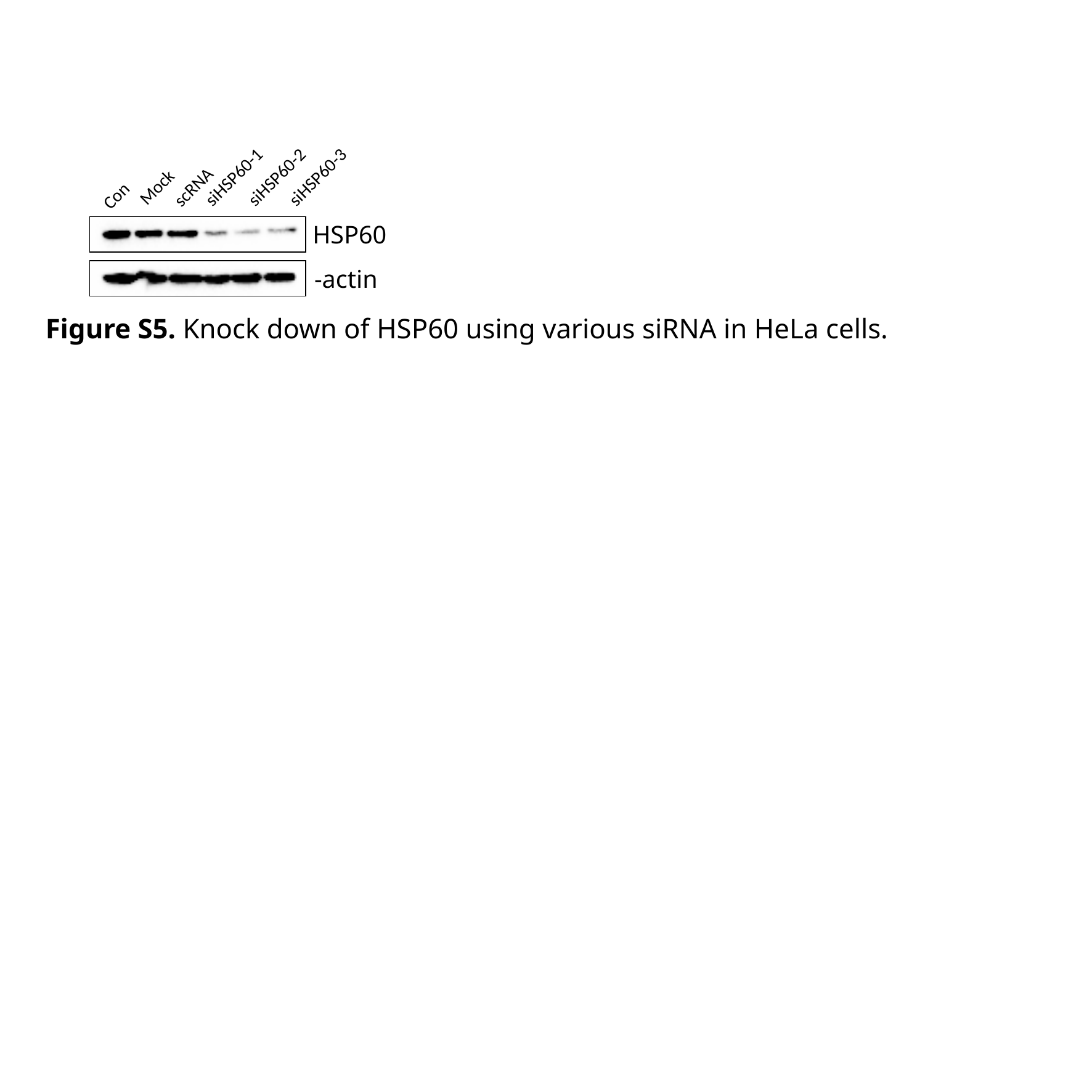

siHSP60-1
siHSP60-2
siHSP60-3
Mock
scRNA
Con
HSP60
Figure S5. Knock down of HSP60 using various siRNA in HeLa cells.

## Slide 13
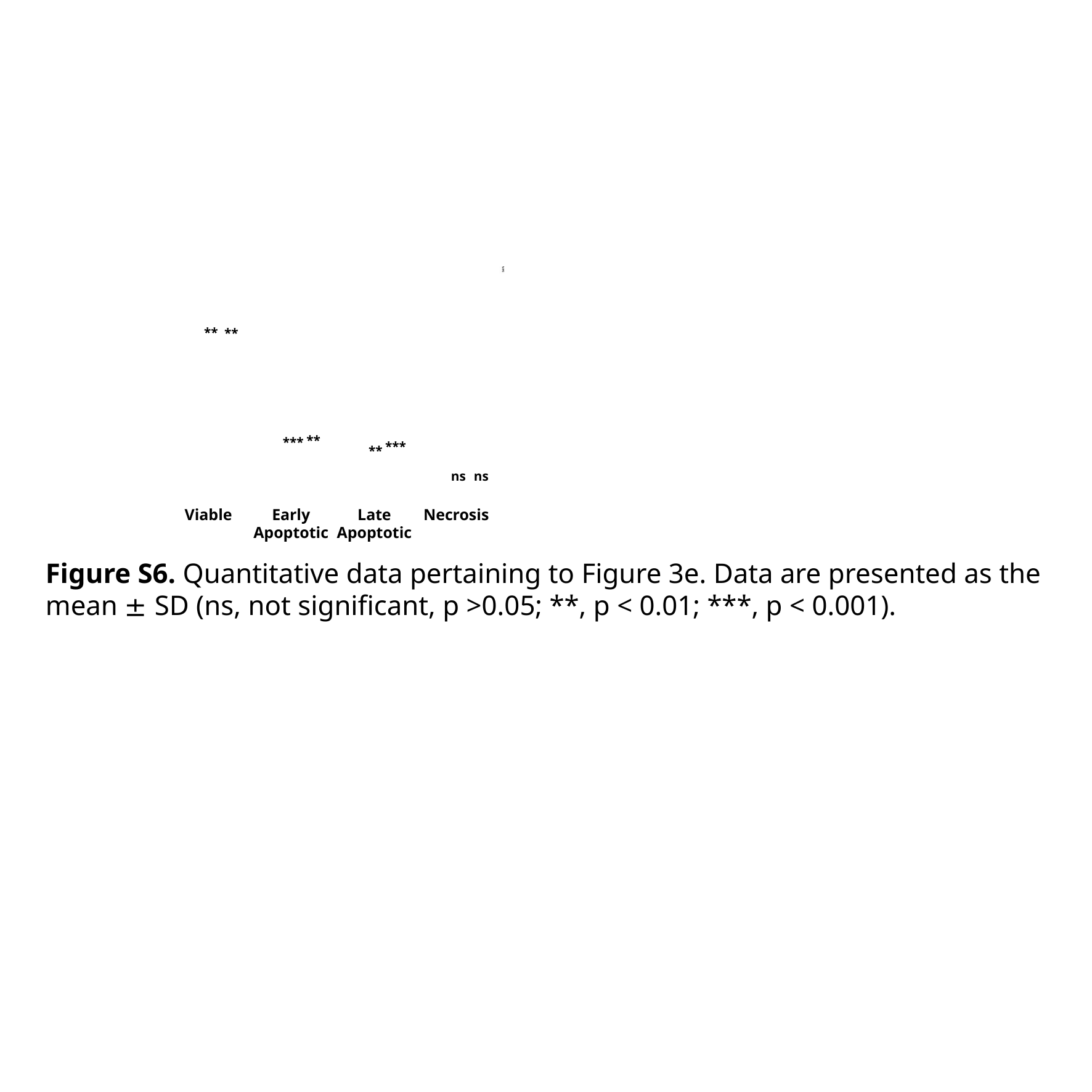

**
**
**
***
***
**
ns
ns
Viable
Early
Apoptotic
Late
Apoptotic
Necrosis
Figure S6. Quantitative data pertaining to Figure 3e. Data are presented as the mean ± SD (ns, not significant, p >0.05; **, p < 0.01; ***, p < 0.001).

## Slide 14
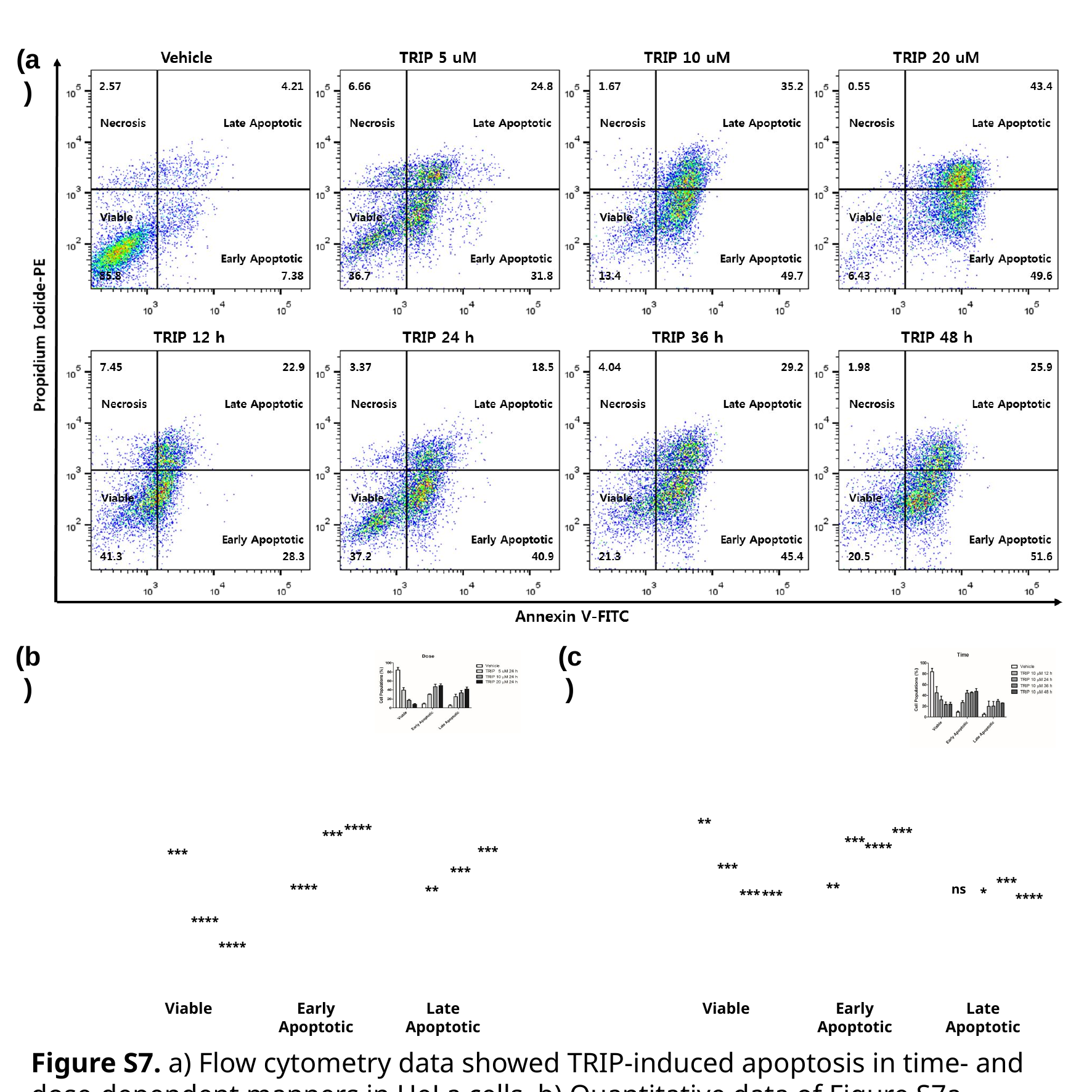

(a)
(b)
(c)
**
***
***
****
***
***
**
ns
*
***
***
****
Viable
Early
Apoptotic
Late
Apoptotic
****
***
***
***
***
****
**
****
****
Viable
Early
Apoptotic
Late
Apoptotic
Figure S7. a) Flow cytometry data showed TRIP-induced apoptosis in time- and dose-dependent manners in HeLa cells. b) Quantitative data of Figure S7a. Data are presented as the mean ± SD (ns, not significant, p >0.05; *, p < 0.05; **, p < 0.01; ***, p < 0.001; ****, p < 0.0001).

## Slide 15
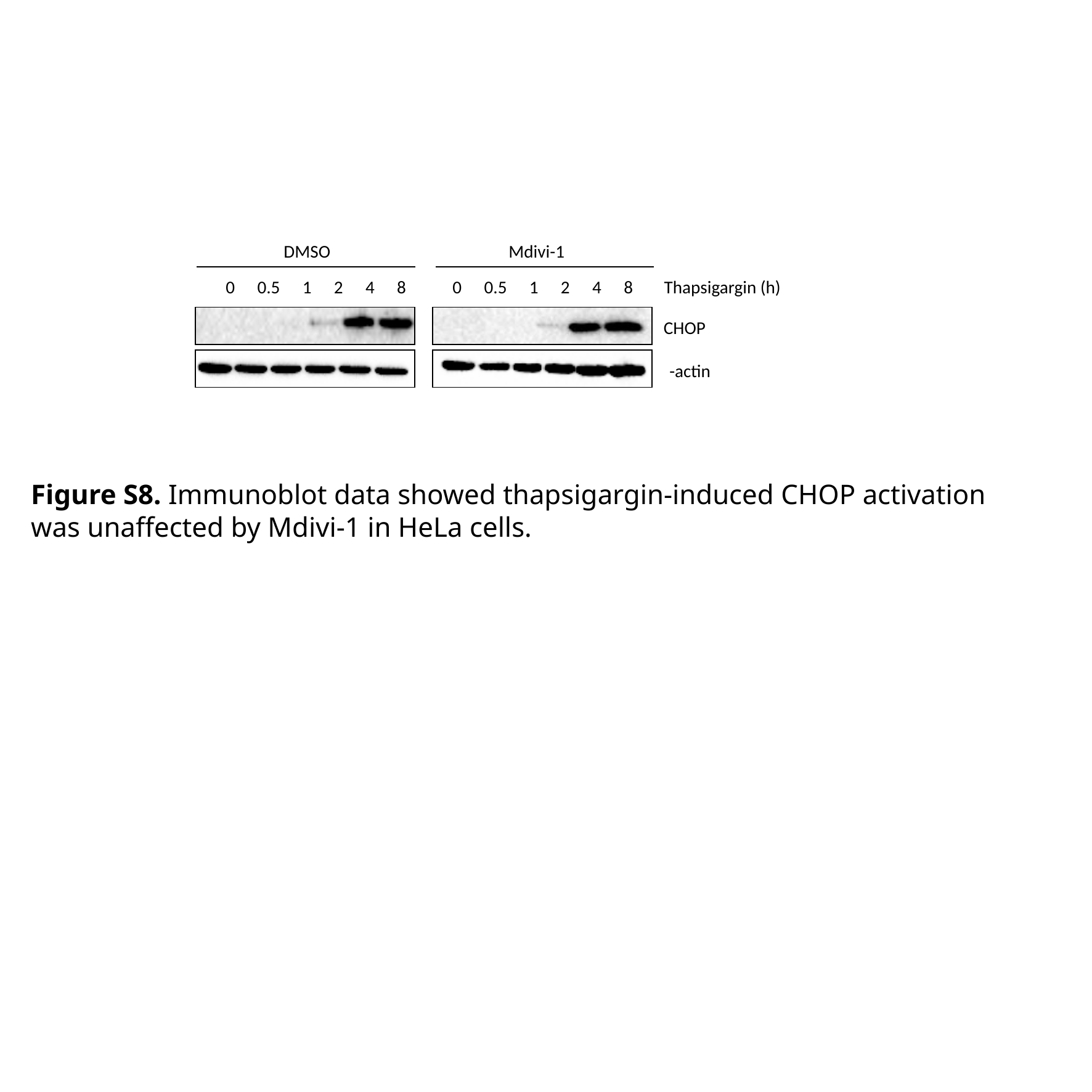

DMSO
Mdivi-1
0
0.5
1
2
4
8
0
0.5
1
2
4
8
Thapsigargin (h)
CHOP
Figure S8. Immunoblot data showed thapsigargin-induced CHOP activation was unaffected by Mdivi-1 in HeLa cells.

## Slide 16
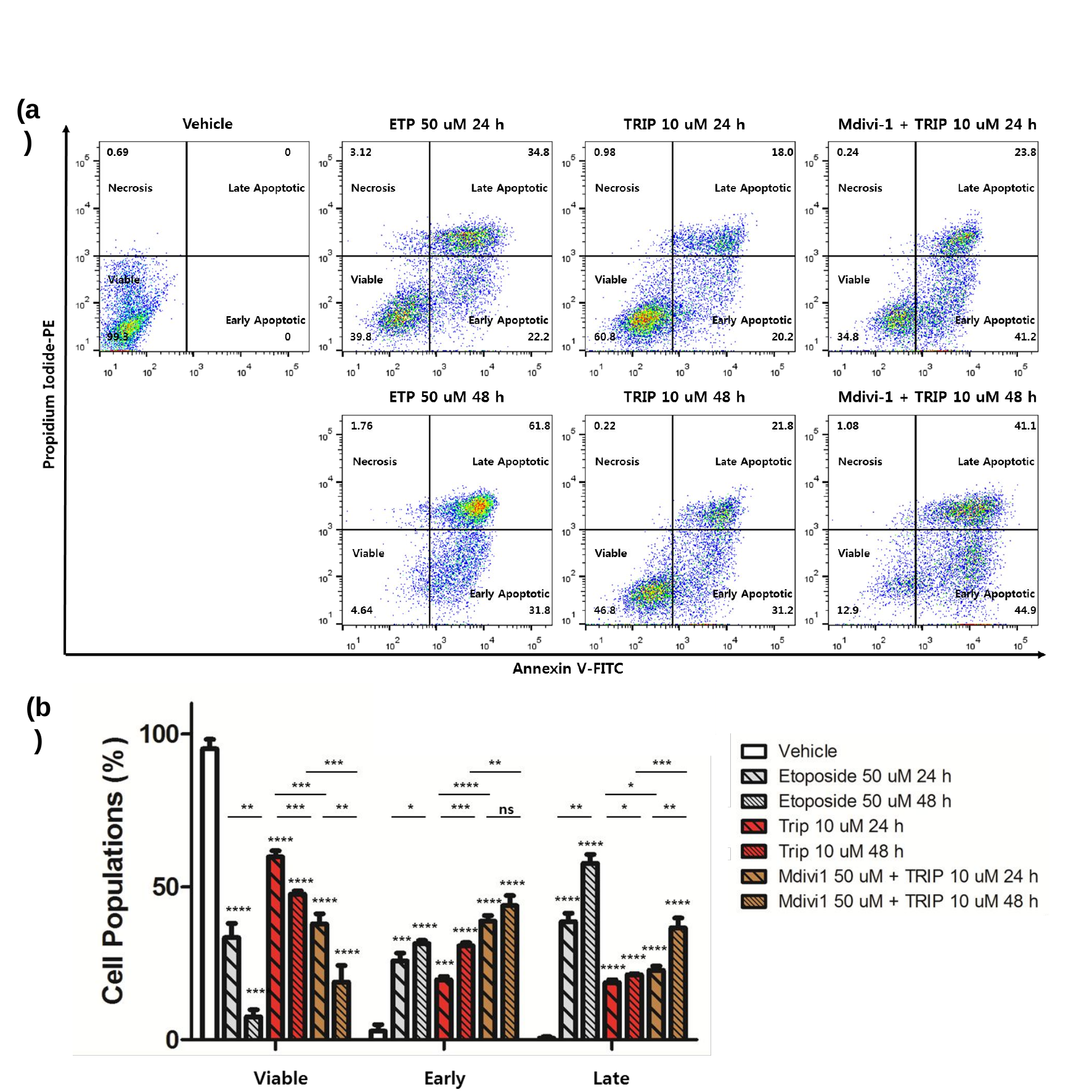

(a)
(b)
Figure S9. a) Flow cytometry data showed CHOP activation was enhanced by 50 μM of Mdivi-1 cotreatment with 10 of μM TRIP in time-dependent manners in HeLa cells. b) Quantitative data pertaining to Figure S9a. Data are presented as the mean ± SD (ns, not significant, p >0.05; *, p < 0.05; **, p < 0.01; ***, p < 0.001; ****, p < 0.0001).

## Slide 17
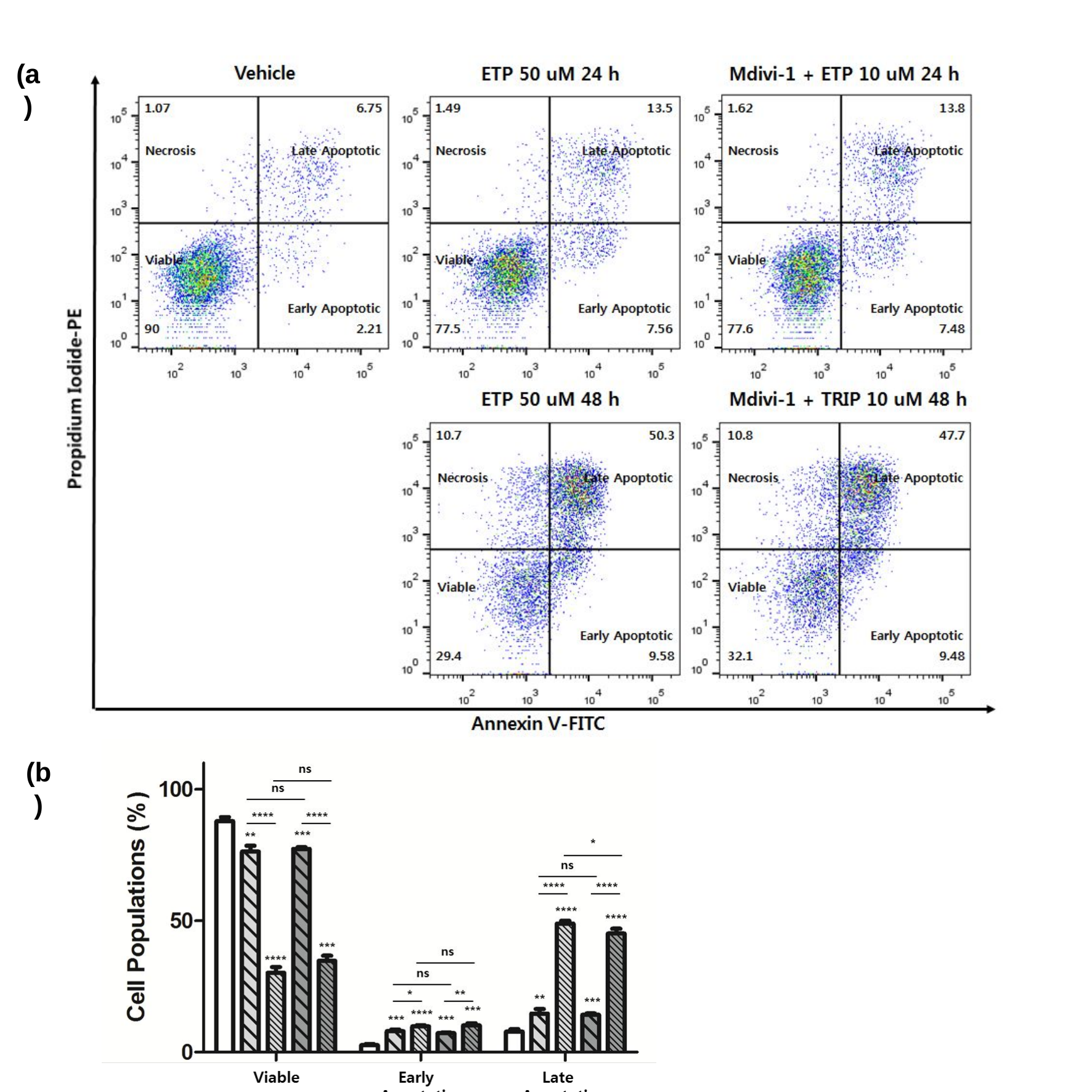

(a)
(b)
Figure S10. a) Flow cytometry data showed apoptosis was unaffected by 50 μM of Mdivi-1 cotreatment with 50 of μM Etoposide in time-dependent manners in HeLa cells. b) Quantitative data pertaining to Figure S10a. Data are presented as the mean ± SD (ns, not significant, p >0.05; *, p < 0.05; **, p < 0.01; ***, p < 0.001; ****, p < 0.0001).

## Slide 18
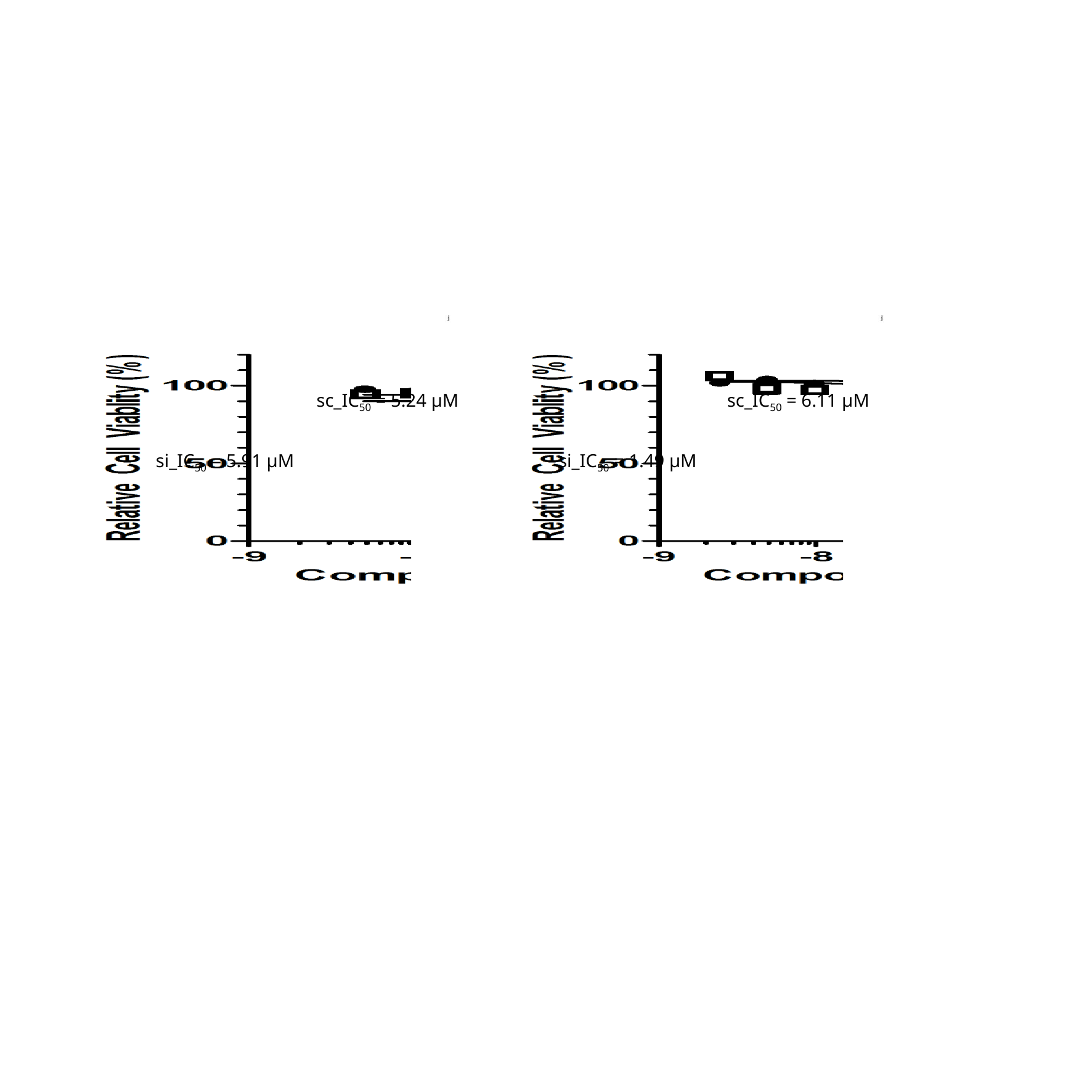

sc_IC50 = 5.24 μM
sc_IC50 = 6.11 μM
si_IC50 = 5.91 μM
si_IC50 = 1.49 μM

## Slide 19
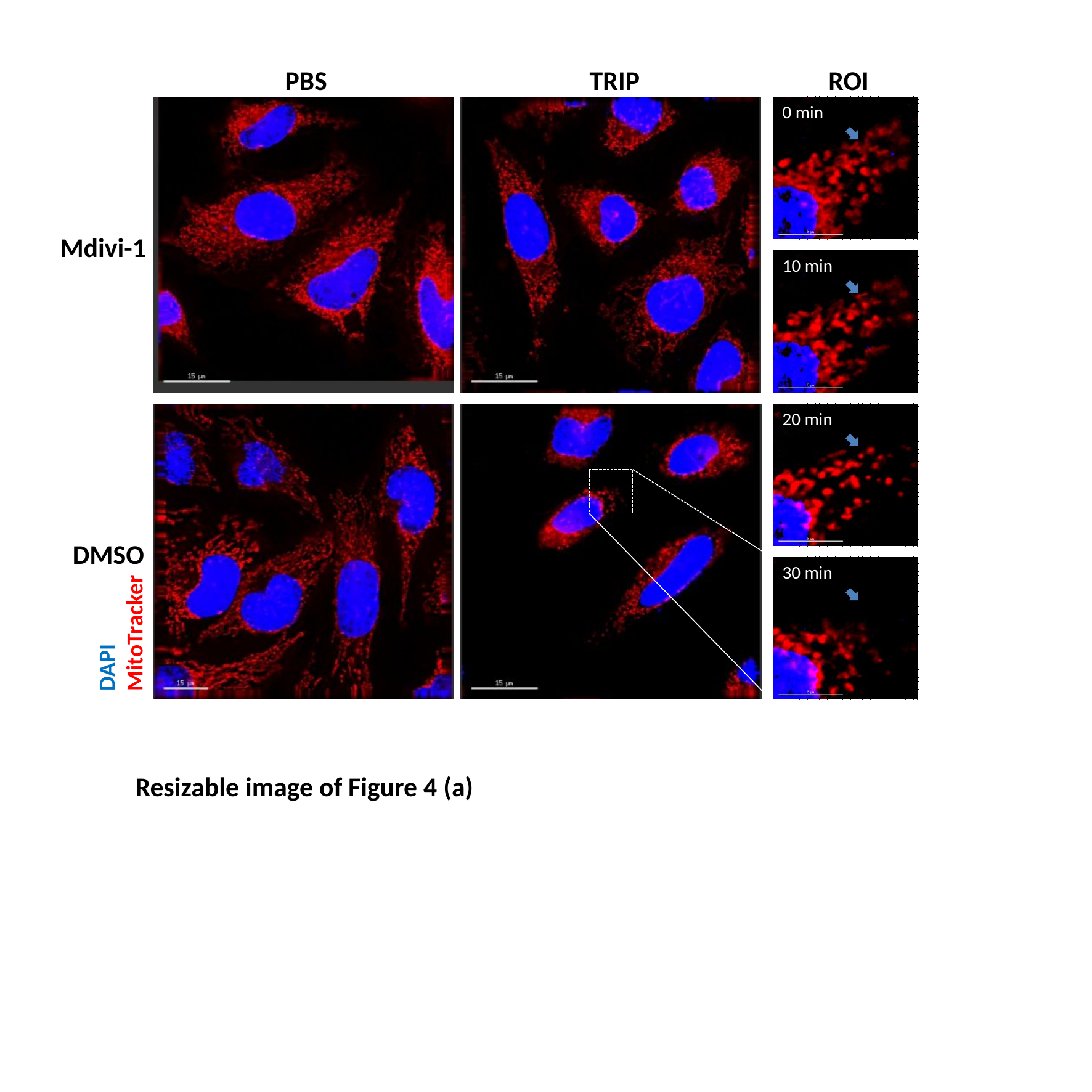

PBS
TRIP
ROI
0 min
Mdivi-1
10 min
20 min
DMSO
30 min
DAPI
MitoTracker
Resizable image of Figure 4 (a)

## Slide 20
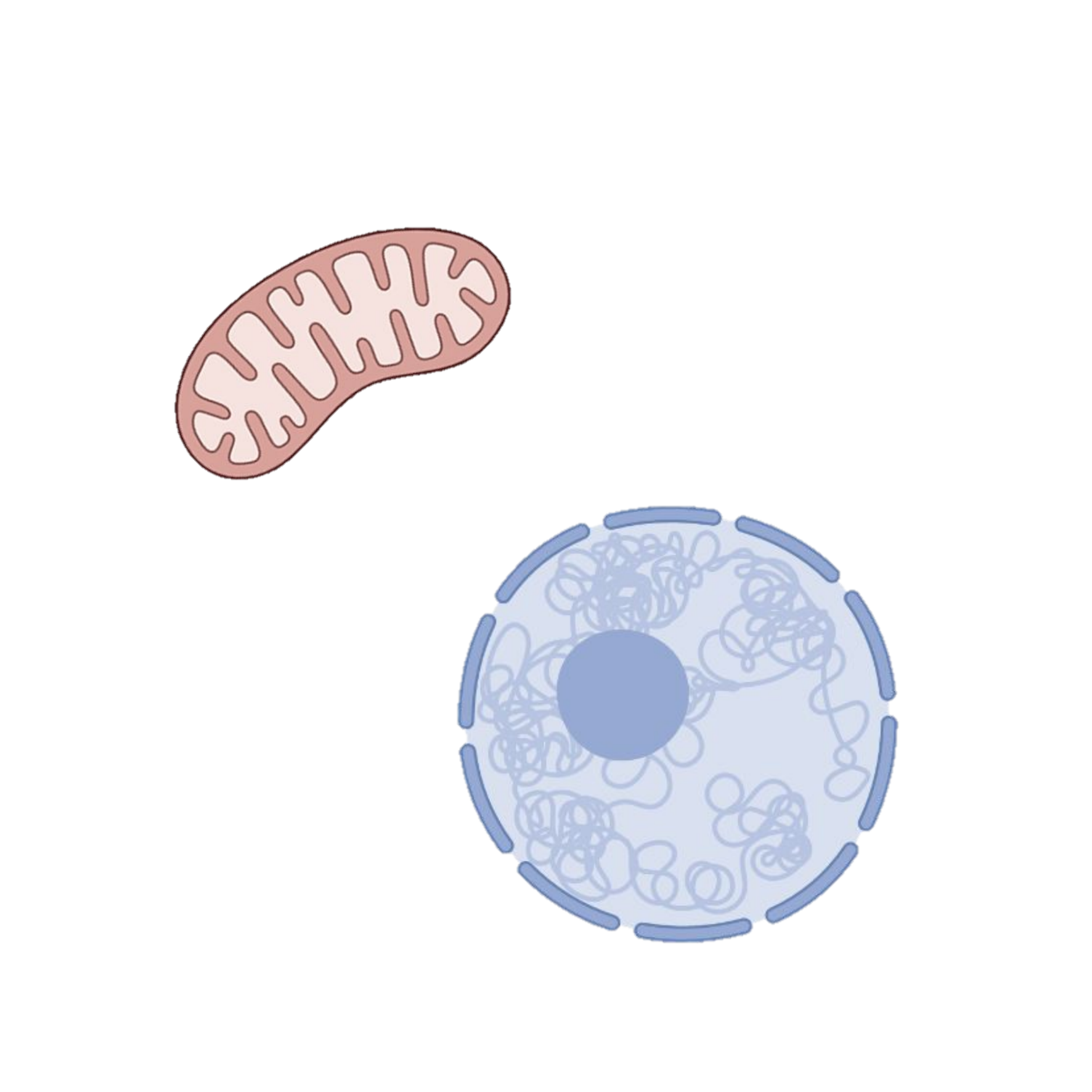

Supplement: Supplementary file 1 [file Presentation1.PPTX]
