# Supplementary material for: Label-Free Target Identification Reveals the Anticancer Mechanism of a Rhenium Isonitrile Complex
Source: Front Chem. 2022 Mar 14;10:850638. doi: 10.3389/fchem.2022.850638 (PMC8964423; doi:10.3389/fchem.2022.850638)
Supplement: Supplementary file 2 [file DataSheet1.pdf]

Supplementary Material

Label-free Target Identification Reveals the Anticancer Mechanism of a Rhenium Isonitrile Complex

Junhyeong Yim[1] and Seung Bum Park[1,2]*

[1]Department of Biophysics and Chemical Biology, Seoul National University, Seoul, Republic of Korea.

[2]CRI Center for Chemical Proteomics, Department of Chemistry, Seoul National University, Seoul, Republic of Korea

*** Correspondence:**
Prof. Seung Bum Park
sbpark@snu.ac.kr

1 Supplementary Figures

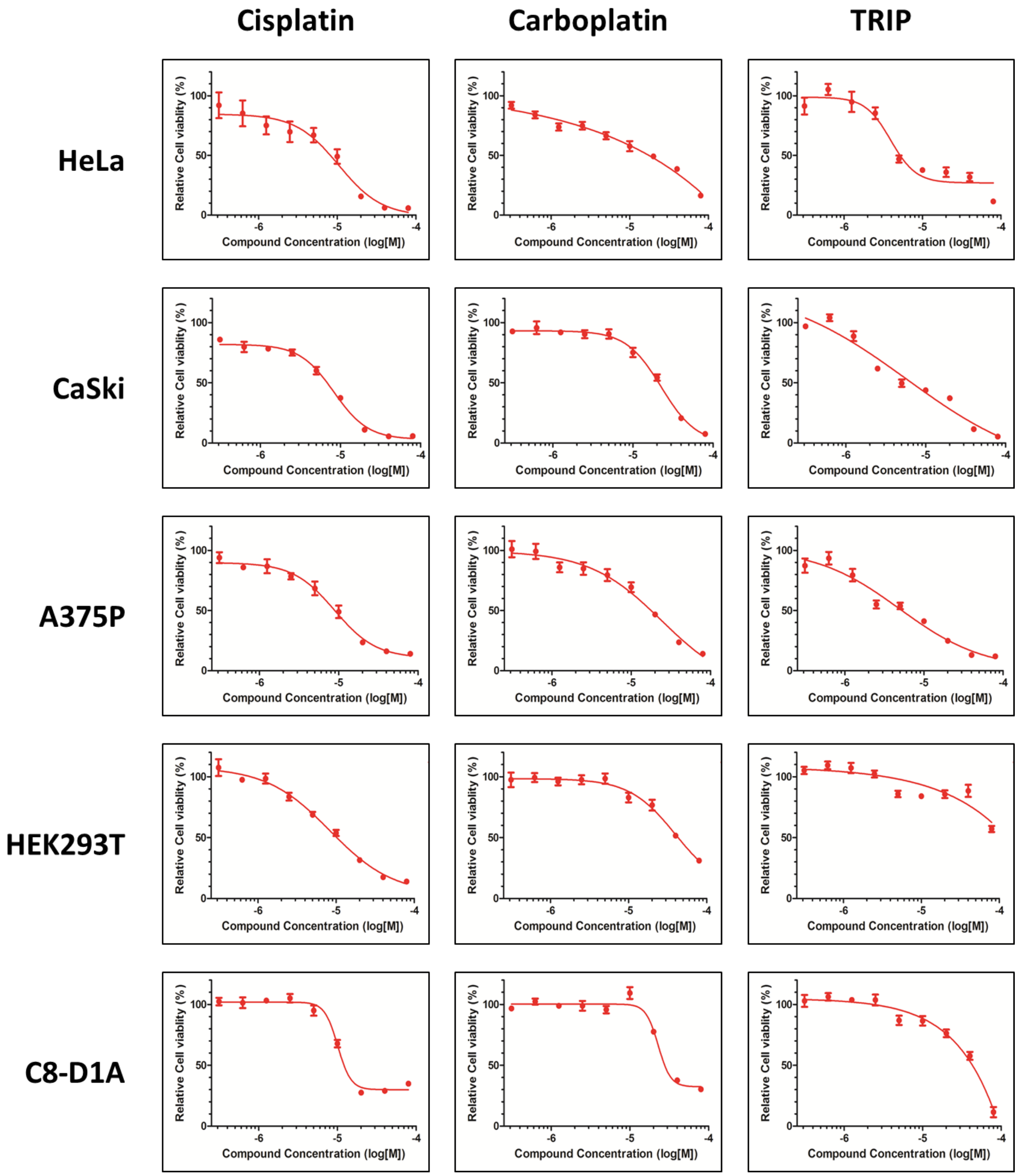

Figure S1. Dose-response curves of cisplatin, carboplatin, and TRIP in various cell lines (n=6). These data are summarized in Table 1. Data are presented as the mean ± SD.

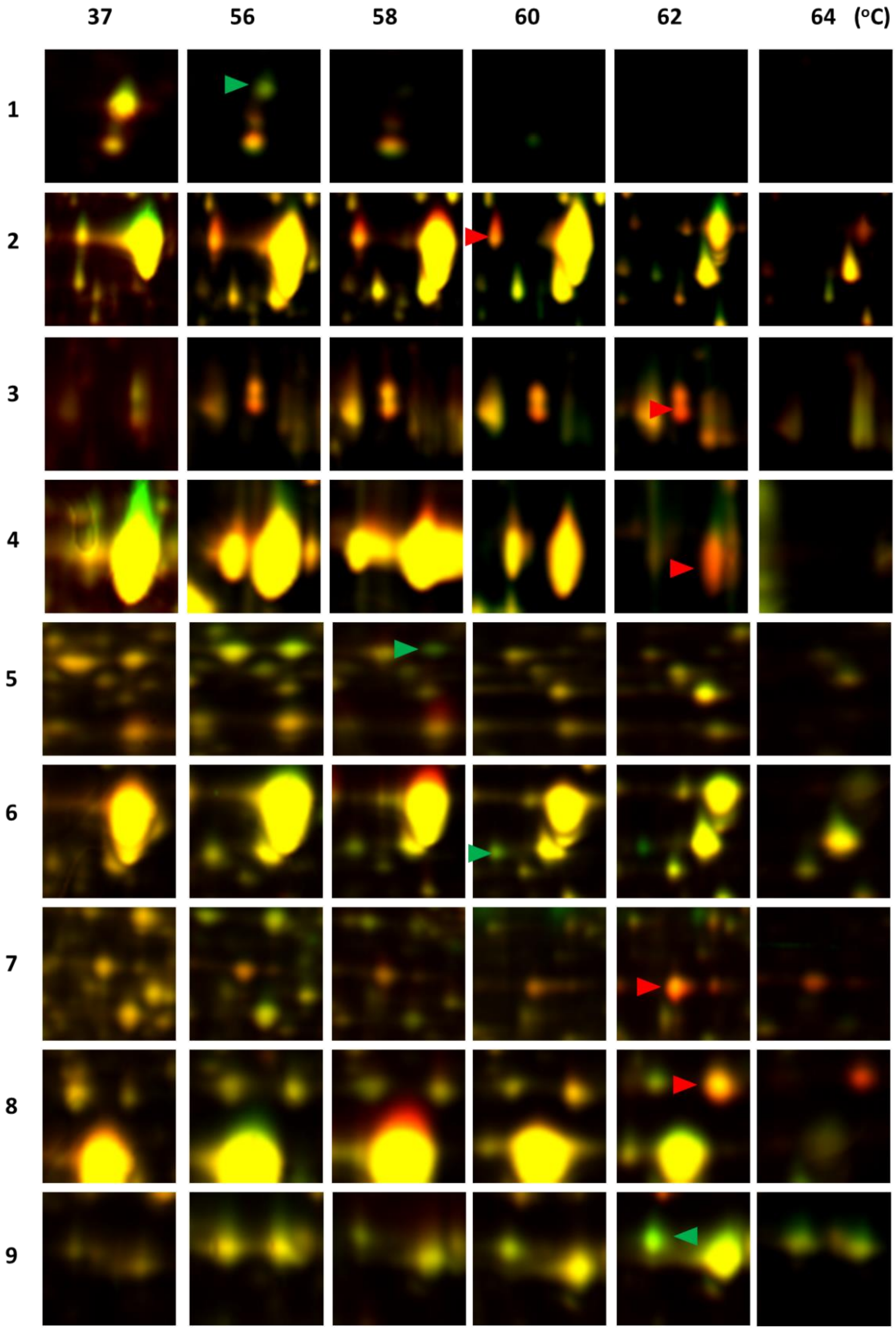

Figure S2. Target identification of TRIP using TS-FITGE revealed nine protein spots, including heat-sensitive green spots and heat-resistant red spots in 2-D gels.

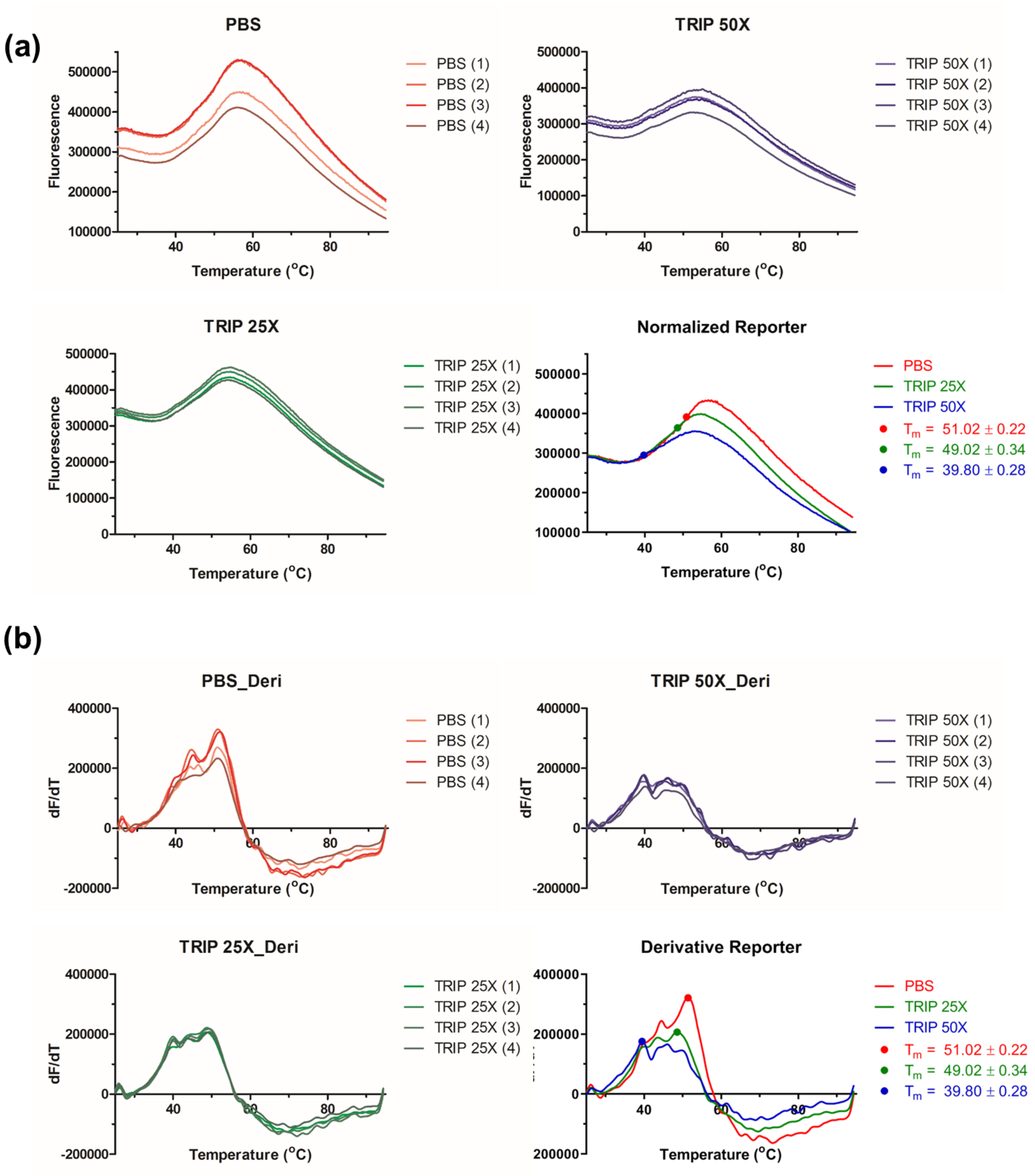

Figure S3. Total data of melting curves (a) and first derivatives (b) of differential scanning fluorimetry (DSF) analysis (n=4).

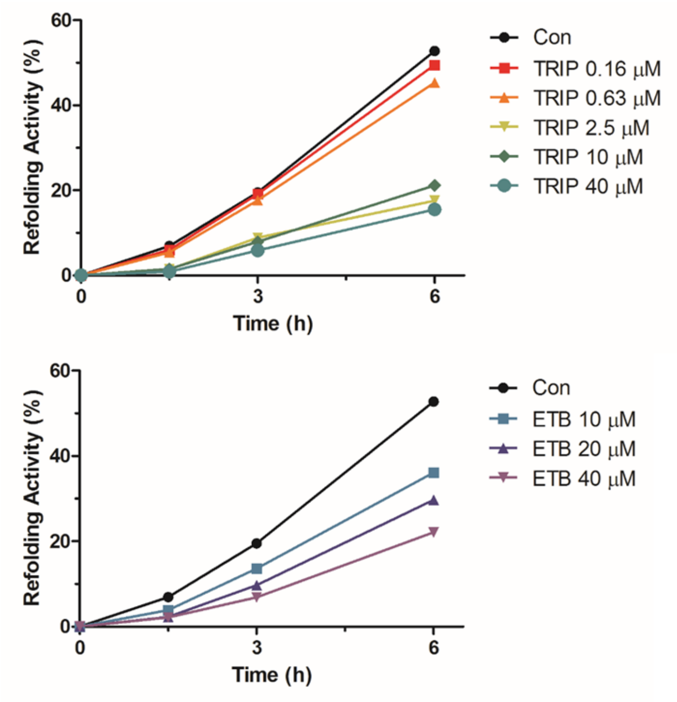

Figure S4. HSP60/HSP10 refolding assay data. A complete data set of Figure 2e. Heat-denatured proteins were refolded by HSP60/HSP10 heterodimer in the absence or presence of either TRIP or epolactaene *t*-butyl ester (ETB) for 1.5, 3, and 6 h.

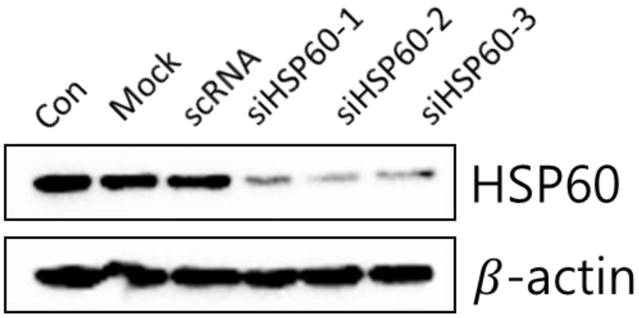

Figure S5. Knockdown of HSP60 using various siRNAs in HeLa cells.

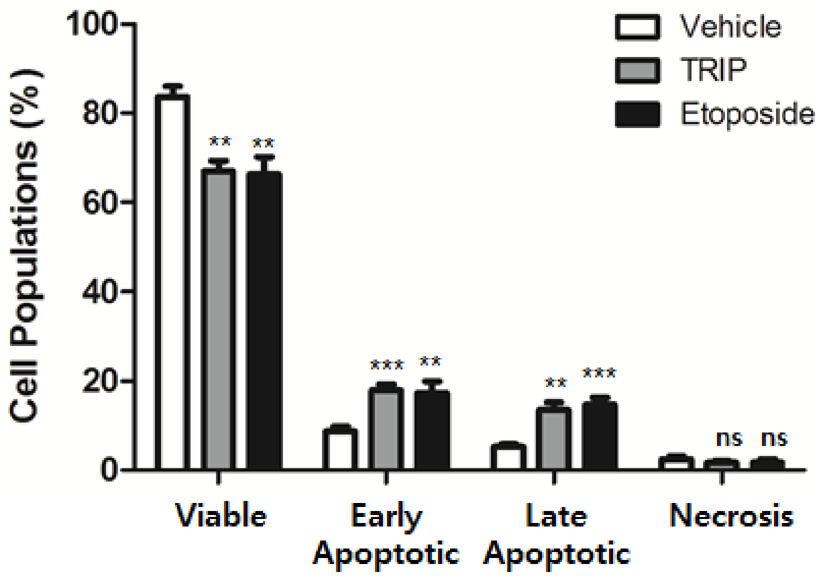

Figure S6. Quantitative data pertaining to Figure 3e (n=3). Data are presented as the mean ± SD (ns, not significant, $p > 0.05$; **, $p < 0.01$; ***, $p < 0.001$).

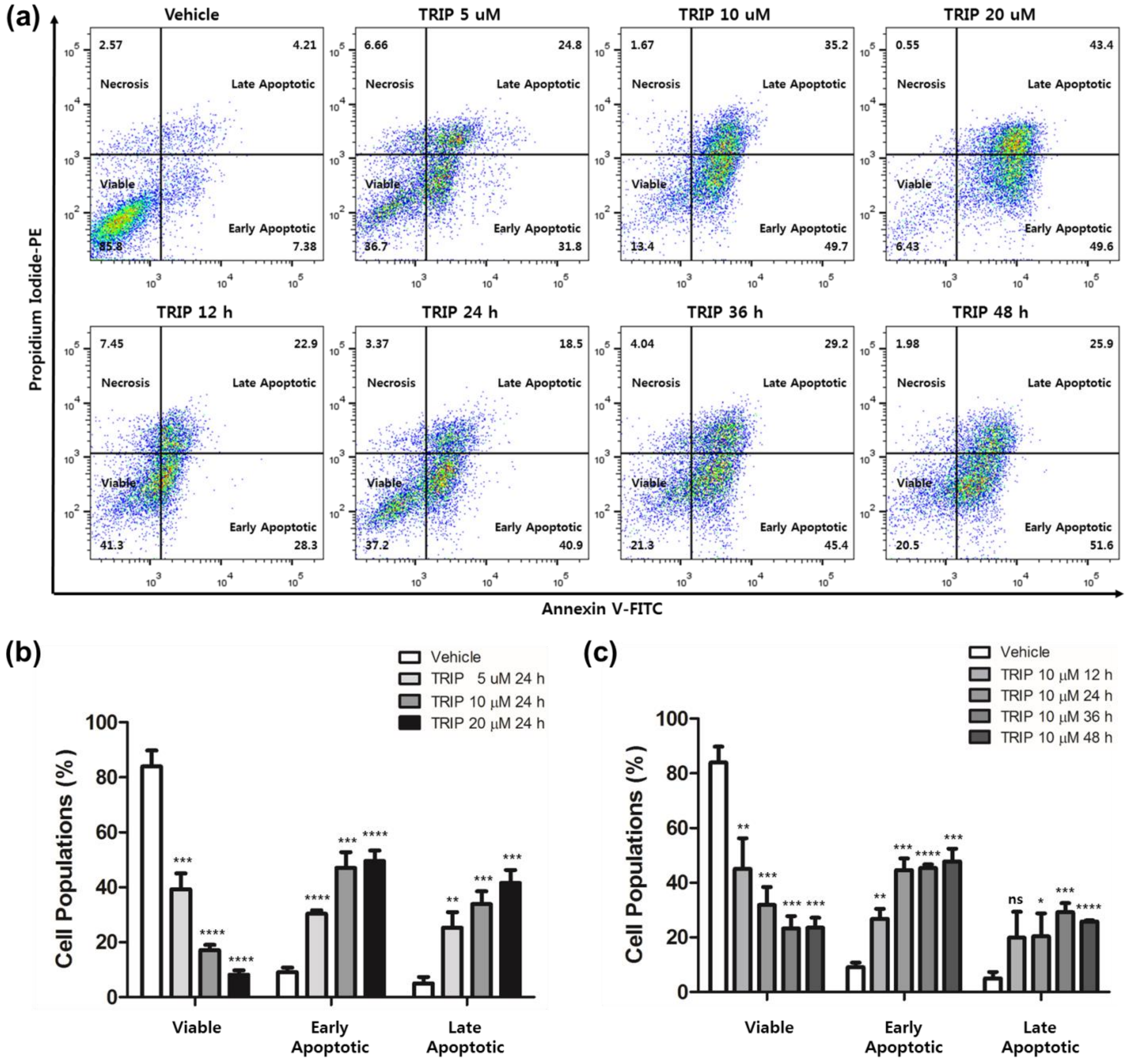

Figure S7. (a) Flow cytometry data showed TRIP-induced apoptosis in time- and dose-dependent manners in HeLa cells. (b) Quantitative data of Figure S7a (n=3). Data are presented as the mean ± SD (ns, not significant, $p > 0.05$; *, $p < 0.05$; **, $p < 0.01$; ***, $p < 0.001$; ****, $p < 0.0001$).

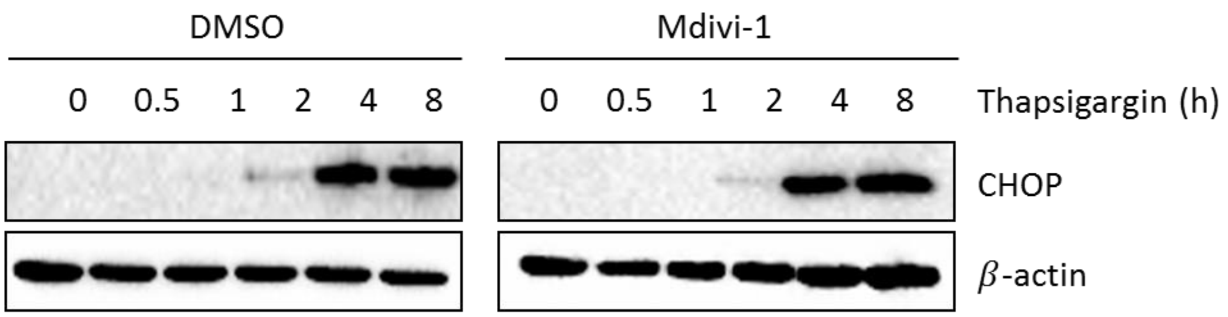

Figure S8. Immunoblot data showed that thapsigargin-induced CHOP activation was unaffected by Mdivi-1 treatment in HeLa cells.

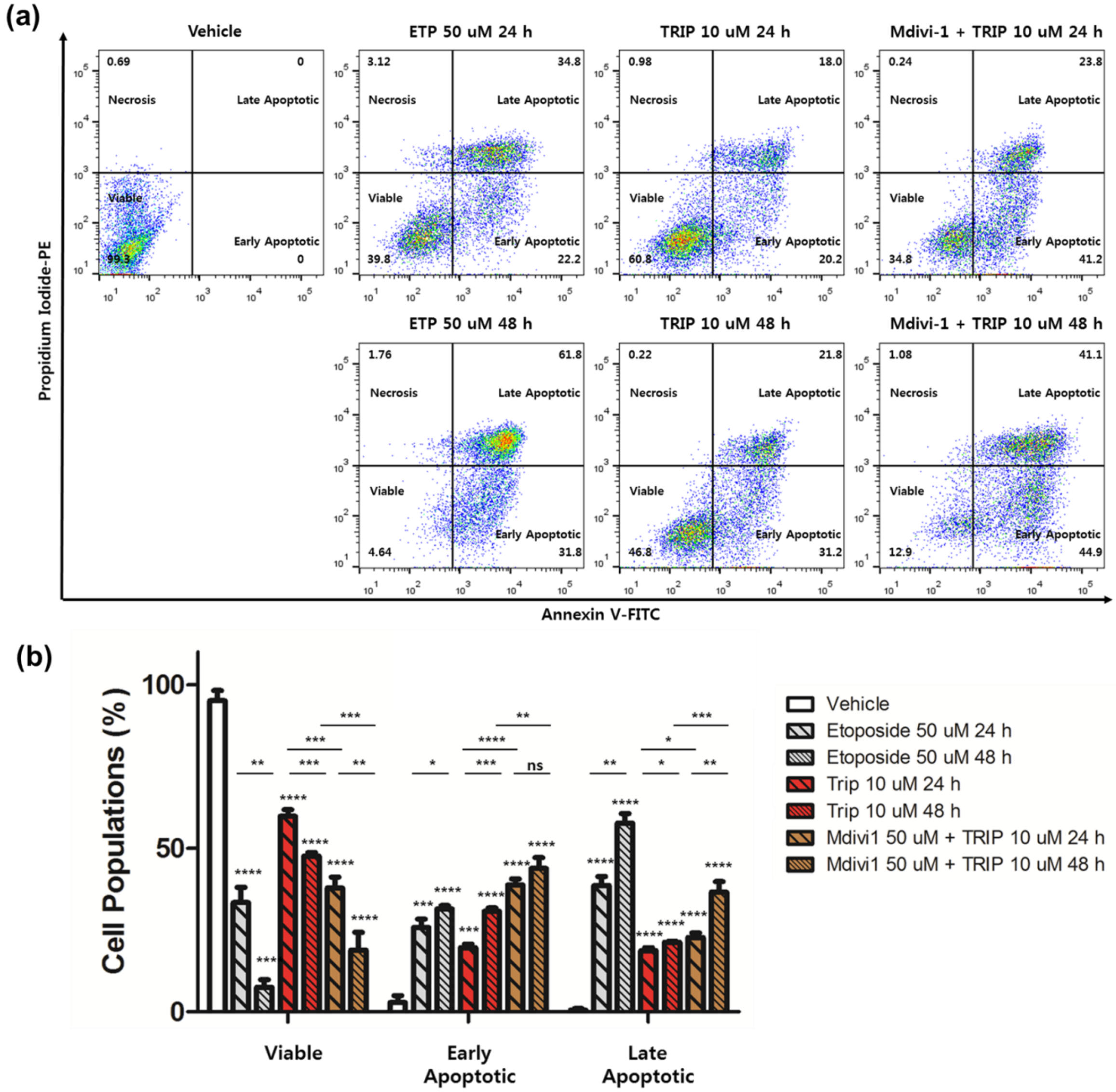

Figure S9. (a) Flow cytometry data showed that apoptosis in HeLa cells was enhanced by 50 µM of Mdivi-1 co-treatment with 10 µM of TRIP in a time-dependent manner. (b) Quantitative data pertaining to Figure S9a (n=3). Data are presented as the mean ± SD (ns, not significant, p >0.05; *, p < 0.05; **, p < 0.01; ***, p < 0.001; ****, p < 0.0001).

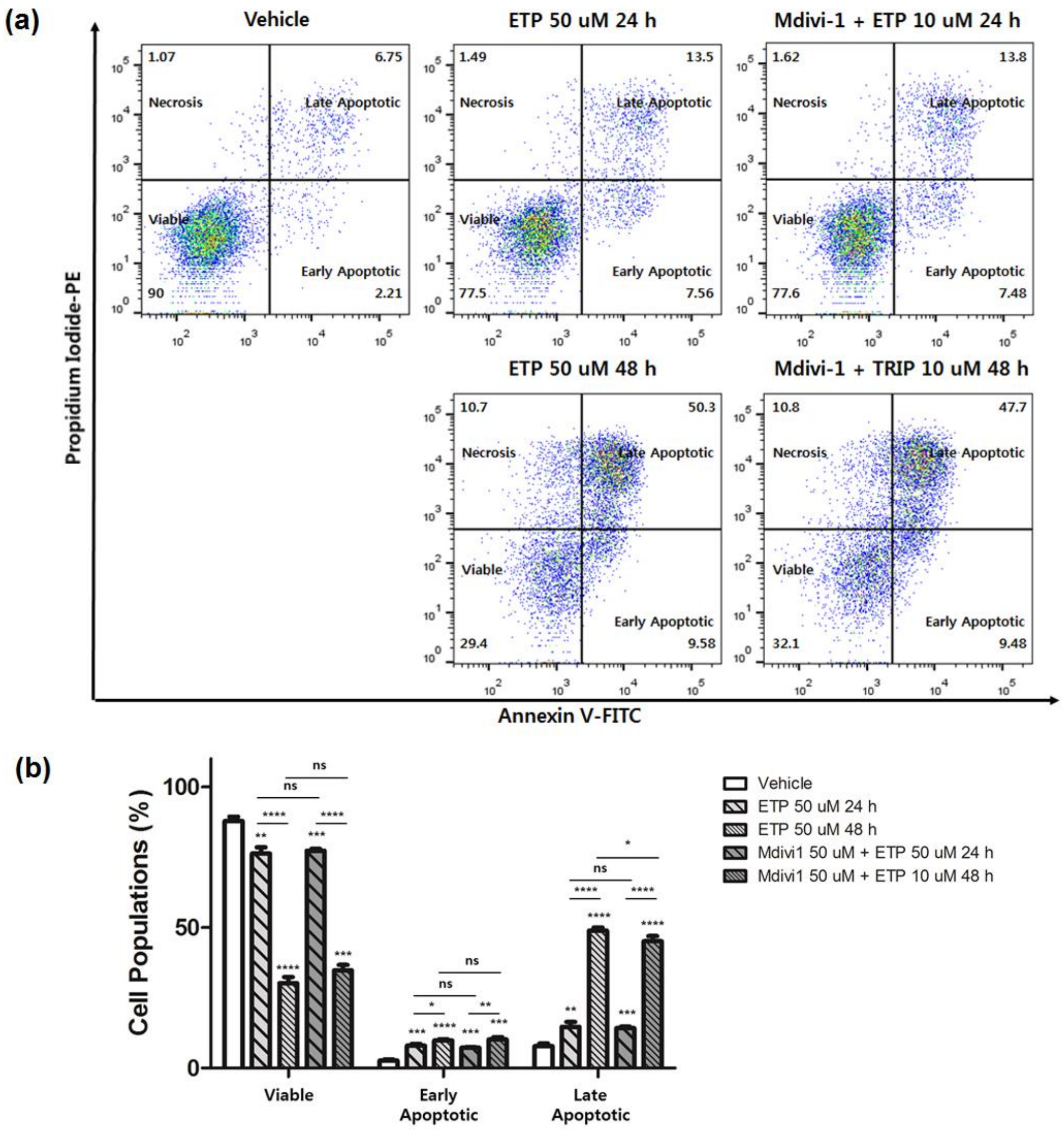

Figure S10. (a) Flow cytometry data showed that apoptosis in HeLa cells was unaffected by 50 µM of Mdivi-1 co-treatment with 50 µM of Etoposide in a time-dependent manner. (b) Quantitative data pertaining to Figure S10a (n=3). Data are presented as the mean ± SD (ns, not significant, p >0.05; *, p < 0.05; **, p < 0.01; ***, p < 0.001; ****, p < 0.0001).

2 Supplementary Table

Table S1. Mass spectrometry results of the observed nine spots in TS-FITGE.

Spot No.	Match to	Molecular Weight	Mascot Score	Queries matched	Sequence Coverage (%)	Protein
1	GSTP1_HUMAN	23341	765	40	60	Glutathione S-transferase P
	PARK7_HUMAN	19878	117	9	48	Protein DJ-1
	RB11A_HUMAN	24378	101	7	31	Ras-related protein Rab-11A
2	ENOA_HUMAN	47139	1919	114	68	Alpha-enolase
	ENO1B_HUMAN	49446	661	31	20	Alpha-enolase, lung specific
	LA_HUMAN	46808	136	10	22	Lupus La protein
3	RLA0_HUMAN	34252	760	79	70	60S acidic ribosomal protein P0
	LDHB_HUMAN	36615	101	7	20	L-lactate dehydrogenase B chain
	IPYR2_HUMAN	37896	99	6	18	Inorganic pyrophosphatase 2, mitochondrial precursor
4	CH60_HUMAN	61016	3093	297	71	60 kDa heat shock protein, mitochondrial precursor
	ACTG_HUMAN	41766	303	25	39	Actin, cytoplasmic 2
5	IMDH2_HUMAN	55770	425	59	35	Inosine-5'-monophosphate dehydrogenase 2
	G6PD_HUMAN	59219	216	23	33	Glucose-6-phosphate 1-dehydrogenase
	PCCB_HUMAN	58179	142	11	22	Propionyl-CoA carboxylase beta chain, mitochondrial precursor
	SERA_HUMAN	56614	109	9	21	D-3-phosphoglycerate dehydrogenase
6	IDHC_HUMAN	46630	2545	168	72	Isocitrate dehydrogenase [NADP] cytoplasmic
	EFTU_HUMAN	49510	192	13	27	Elongation factor Tu, mitochondrial precursor
	PUR6_HUMAN	47049	178	15	33	Multifunctional protein ADE2
	MDHC_HUMAN	36403	124	7	20	Malate dehydrogenase, cytoplasmic
7	TRXR1_HUMAN	54672	1256	129	54	Thioredoxin reductase 1, cytoplasmic precursor

	CH60_HUMAN	61016	134	10	26	60 kDa heat shock protein, mitochondrial precursor
	PRS4_HUMAN	49154	110	7	12	26S protease regulatory subunit 4
8	DHE3_HUMAN	61359	2309	199	60	Glutamate dehydrogenase 1, mitochondrial precursor
	ATPA_HUMAN	59714	268	14	22	ATP synthase subunit alpha, mitochondrial precursor
	PUR8_HUMAN	54854	117	6	12	Adenylosuccinate lyase
9	ATPA_HUMAN	59714	2555	176	51	ATP synthase subunit alpha, mitochondrial precursor
	GLYM_HUMAN	55958	194	12	21	Serine hydroxymethyltransferase, mitochondrial precursor
	GSHR_HUMAN	56221	167	7	15	Glutathione reductase, mitochondrial precursor
	DNPEP_HUMAN	52395	125	5	17	Aspartyl aminopeptidase
